# Preparation of Multifunctional Alginate–PEG–Chitosan Double Shell and Thyme Oil–Oleic Acid Core Microcapsules via Coaxial Electrospraying

**DOI:** 10.3390/polym18172082

**Published:** 2026-08-27

**Authors:** Emel Onder, Sena Saritop, Nihal Sarier

**Affiliations:** 1 Faculty of Textile Technologies and Design, Istanbul Technical University, Gumussuyu, Istanbul 34437, Türkiye; 2 Faculty of Engineering, Istanbul Kültür University, Atakoy Campus, Bakirkoy, Istanbul 34156, Türkiye; 3 Spectroscopy@IKU, IKU-SPECTRA Molecular Sciences and Spectroscopy Applied Research Center, Istanbul Kültür University, Atakoy Campus, Bakırkoy, Istanbul 34156, Türkiye

**Keywords:** alginate, chitosan, PEG, thyme oil, coaxial electrospraying, heat storage

## Abstract

The growing interest in bio-based and bioactive materials, as well as sustainable production techniques, has driven the development of multifunctional hybrid systems. This study reports the fabrication of novel microcapsules with a double-layer alginate–PEG–chitosan shell, with or without a core, via coaxial electrospraying, followed by ionotropic gelation and polyelectrolyte complexation. PEG1000 and PEG1500 were incorporated into the shell as phase change materials, and thyme oil–oleic acid served as a hydrophobic bioactive core. Scanning electron microscopy and Fourier transform infrared analyses confirmed the structural integrity and effective shell–core integration. Thermogravimetric analyses showed enhanced thermal stability in double-layer alginate–PEG–chitosan biopolymer network shell and thyme oil included core system, with a delayed degradation up to 370.0 °C and reduced mass loss compared to the alginate–chitosan control sample. Differential scanning calorimetry over ten heating–cooling cycles demonstrated significant phase transition enthalpies (70.5–91.8 J·g^−1^ at 37.6–48.5 °C), confirming efficient thermal energy storage and release governed by the PEG content. Aqueous suspensions prepared from microcapsules exhibited reversible temperature-dependent swelling–deswelling behavior between 20.0 and 55.0 °C, governed by hydrogel properties of the alginate–chitosan shell interactions. The microcapsules exhibited pronounced pH-dependent swelling (enhanced at pH 7.0), high water solubility, and good antioxidant activity. These findings highlight the broad application potential of bio-based shell–core microcapsules, e.g., active food packaging, biomedical dressings, protective coatings, pharmaceutical and biomedical delivery systems and functional textiles.

## 1. Introduction

The utilization of bio-based and bioactive substances in advanced functional products across diverse industries, alongside the implementation of environmentally sustainable production techniques, has increased substantially in recent years, accompanied by growing scientific and industrial interest in these materials. Microencapsulation is a widely employed technique in which substances are enclosed within a protective matrix at the microscale, enabling enhanced stability, controlled release, and functional performance across diverse applications in pharmaceutical [1,2], food [3], cosmetic [4], textile [5], biomedical [6], and agricultural industries [7,8]. Recently, coaxial electrospraying has gained attention as an advanced microencapsulation technique that enables the fabrication of shell–core microcapsules by simultaneously delivering two immiscible liquid phases through a coaxial needle to produce structured droplets in a high electric field. The applied electrostatic forces induce the formation of a compound Taylor cone, from which a stable shell–core jet emerges and disintegrates into uniformly structured droplets via Rayleigh instability, yielding microcapsules with narrow size distributions [9]. By adjusting parameters such as the voltage and flow rate, the size and morphology of the resulting microcapsules can be precisely controlled. Shell–core electrospraying has several advantages over conventional encapsulation techniques. It can create microcapsules with high encapsulation efficiency, structural integrity, and physicochemical stability. It also does not require thermal input, which is particularly well-suited for encapsulating thermally sensitive bioactive compounds, such as drugs and essential oils.

Sodium alginate, an anionic polysaccharide obtained from brown algae, comprises β-D-mannuronic acid (M) and α-L-guluronic acid (G) units linked by 1,4-glycosidic bonds. Its ability to form hydrogels via ionotropic cross-linking with divalent cations (e.g., Ca^2+^) makes it particularly suitable for encapsulating bioactive molecules. When alginate droplets are introduced into a Ca^2+^ bath, rapid gelation forms a mechanically stable hydrogel shell that limits premature diffusion and evaporation of the encapsulated core. In addition to these properties, alginate is biocompatible, biodegradable, and non-toxic, which supports its use in the protective microencapsulation of bioactive agents with increasing interest [10]. For example, it has been used in the encapsulation and controlled release of oral, topical, and targeted drugs [11], as well as nutraceuticals such as easily degradable betacyanins [12], anti-inflammatories [13], and antioxidants [14,15]. However, alginate-only capsules may suffer from limited long-term stability and active compound leakage. Multilayer encapsulation approaches have been shown to greatly enhance structural integrity, protection efficiency, and sustained release [16,17].

**Figure 1:**
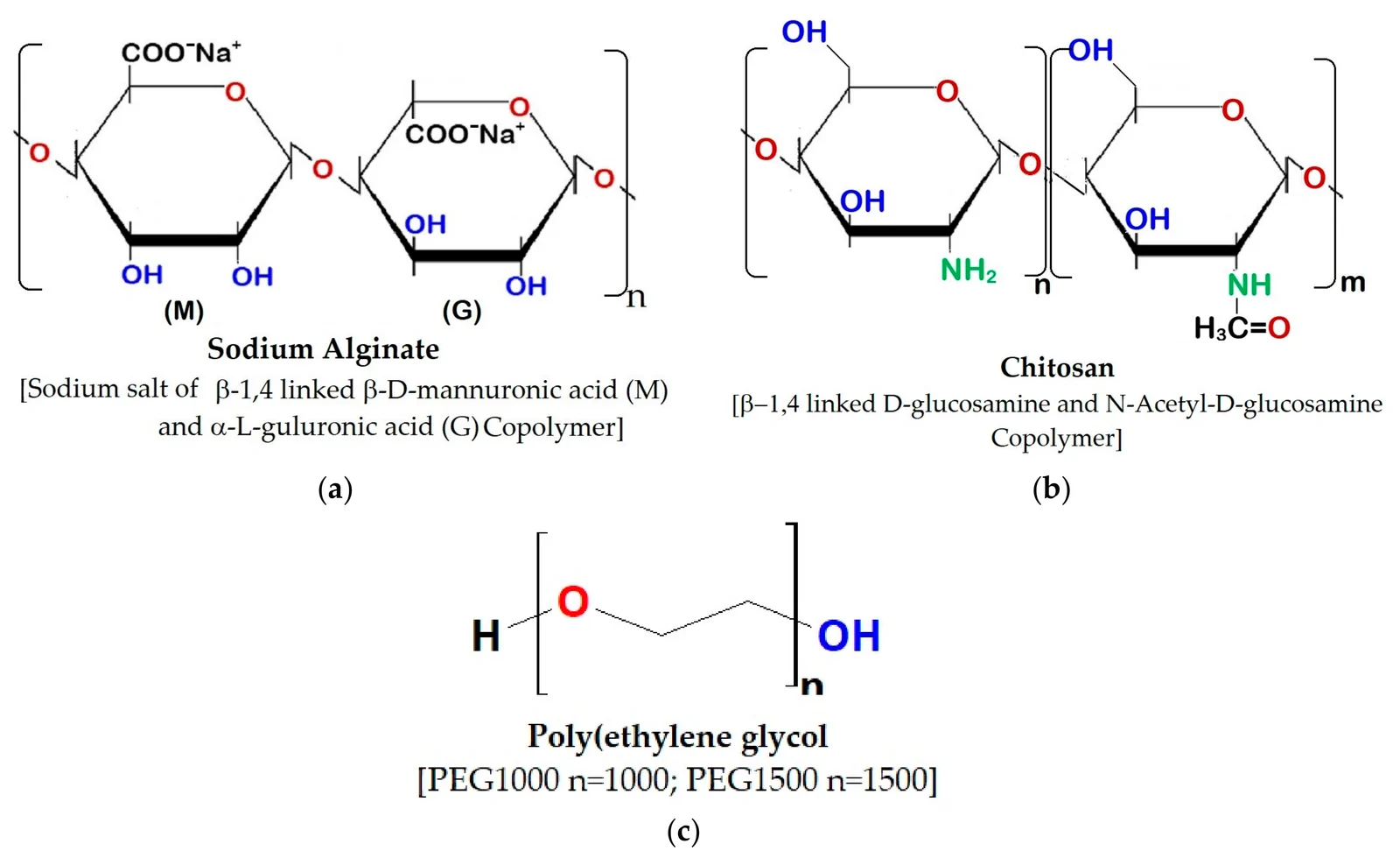
Skeletal structures of shell materials: (**a**) alginate; (**b**) chitosan; (**c**) poly (ethylene glycol).

Chitosan is a polysaccharide produced mainly by the alkaline deacetylation of chitin, which is the most abundant polysaccharide in nature after cellulose. Structurally, chitosan is a linear polymer composed of D-glucosamine and N-acetyl-D-glucosamine units joined by β-(1-4)-glycosidic bonds (Figure 1) [18]. Under slightly acidic conditions, chitosan has a cationic structure that allows it to interact with negatively charged surfaces and anionic polymers. To increase stability and improve permeability, chitosan has been employed as a coating for calcium alginate beads, which forms a strong complex membrane via electrostatic interactions between positively charged amino (–NH_2_) groups of chitosan and negatively charged carboxyl (–COO^−^) and hydroxyl (–OH^−^) groups of alginates, thereby forming a uniform polyelectrolyte complex layer [19,20]. The formation of this secondary layer of chitosan depends on the chitosan molecular weight, concentration, pH of the solution, and immersion time, which can be optimized to customize the thickness, permeability, and surface properties of the beads [21,22].

Essential oils (EOs) are volatile plant-derived secondary metabolites composed mainly of terpenoids, phenols, alcohols, and other bioactive constituents. This diverse phytochemical composition underlies their broad biological activities, including antimicrobial, antioxidant, anti-inflammatory, antifungal, and insecticidal effects [23,24]. Owing to these properties, EOs are widely utilized in pharmaceutical, food, cosmetic, agricultural, textile, and functional material applications [25]. Among them, thyme essential oil (*Thymus vulgaris* L.) (TO) is extensively applied in food, pharmaceutical, and flavoring industries. The primary bioactive constituents of thyme essential oil (TO) are thymol, carvacrol, p-cymene, γ-terpinene, terpinen-4-ol, α-terpinolene, linalool, and eucalyptol [26]. Their relative abundance can vary substantially depending on the plant’s genetic background, chemotype, geographical origin, environmental growing conditions, and extraction technique. These compounds exhibit a wide range of biological activities, including antiseptic, antibacterial, antifungal, anthelmintic, antiviral, antioxidant, expectorant, antispasmodic, carminative, diaphoretic, sedative, anti-rheumatic, anti-hyperlipidemic, and anti-hyperglycemic effects [27,28,29]. Thyme oil is classified as generally recognized as safe (GRAS) by the U.S. Food and Drug Administration and is approved as a food flavoring agent by the European Commission [30].

Despite their functional potential, the practical use of EOs is limited by physicochemical limitations as high volatility, poor aqueous solubility, intense aroma, and susceptibility to degradation under environmental stressors such as heat, light, humidity, and oxygen [31,32]. The encapsulation of EOs within polymeric matrices offers several advantages, including protection against degradation, enhancement of stability, prevention of premature release, maintenance of environmental integrity, extension of shelf life, and ensured controlled delivery of active ingredients [24,31,32]. Previous studies have reported the uniaxial electrospray encapsulation of various essential oils, including limonene, oregano, lavender, fennel, and thyme oils [33,34,35,36,37,38,39].

In environments such as food packaging, pharmaceuticals, and cosmetics, thermal buffering is critical for mitigating temperature changes that affect shelf life [1,2,3,4]. Polyethylene glycols (PEGs) are linear polyether polymers with the structure HO–(CH_2_–CH_2_–O)_n_–H. PEGs have gained interest due to their chemical stability, non-toxicity, and molecular-weight-dependent physicochemical properties, including their solid–liquid phase change characteristics. PEG1000 and PEG1500, in particular, exhibit remarkable heat storage/release capacities during reversible phase transitions at moderate temperatures, making them promising phase change materials (PCMs) for improving the thermal buffering performance of polymeric encapsulation systems [40]. Their water and organic solubility enables their efficient incorporation into polymer networks. PEG chains interact with biopolymers like alginate and chitosan through hydrogen bonding between ether oxygen groups and hydroxyl or amino groups [41,42]. These interactions enhance chain mobility, reduce brittleness, and improve structural homogeneity while modulating permeability. Consequently, incorporating PEG into polysaccharide-based shells enhances mechanical stability and strengthens their function as diffusion barrier function, which is particularly beneficial for protecting volatile essential oils [43,44].

Oleic acid (OA) is a monounsaturated fatty acid found in vegetable oils and classified by the FDA as generally recognized as safe (21 CFR 172.860) [45]. In microencapsulation systems, OA functions as a lipophilic surfactant, promoting stable emulsification of EOs and enhancing encapsulation efficiency by reducing interfacial tension between the oil phase and polymeric materials [46,47]. OA has also emerged as a phase change material due to its low melting temperature (4.0–17.0 °C), high latent heat, and chemical stability. OA-based mixtures have been microencapsulated within synthetic polymer shells for thermal energy storage, showing preserved latent heat capacity and reliable thermal cycling performance [48,49].

Current research on the coaxial electrospraying of essential oils using alginate as the primary shell material followed by chitosan coating remains limited. Notably, no published studies have yet incorporated PEG1000 or PEG1500 as phase change materials (PCMs) within the shell. Existing encapsulation approaches for essential oils predominantly utilize single-layer biopolymer matrices or conventional emulsification-based techniques. Although these systems effectively stabilize volatile oils, they generally lack controlled thermal buffering functionality in response to environmental temperature fluctuations, thereby limiting their applicability in active packaging and temperature-sensitive delivery systems.

The present study introduces a novel multifunctional microencapsulation platform based on a double-shell alginate–PEG–chitosan (ALPEG) architecture fabricated via coaxial electrospraying, combining latent heat storage and bioactive functionality within a unique biodegradable system. Unlike conventional PCM microcapsules, which are primarily designed for thermal energy storage using synthetic shell materials, the proposed microcapsules employ a naturally derived alginate–chitosan shell stabilized through sequential Ca^2+^ ionotropic gelation and polyelectrolyte complexation. This environmentally friendly approach eliminates the need for toxic chemical cross-linkers while providing enhanced structural integrity and thermal stability. The successful incorporation of PEG1000 and PEG1500 into the shell matrix, together with the encapsulation of thyme oil–oleic acid in the core, enables simultaneous thermal energy storage–release and natural antioxidant properties. In addition, the pH- and temperature-responsive swelling–deswelling behavior, driven by the intrinsic hydrogel properties of the alginate–chitosan shell, suggests additional opportunities for stimuli-responsive release of encapsulated bioactive compounds, extending the functionality of the system beyond conventional PCM applications. With this aim, six types of ALPEG microcapsules were developed comprising three alginate–PEG–chitosan shell compositions, each prepared either with or without a core. The shell formulations were obtained by including PEG1000 and/or PEG1500 at different mass ratios. The core emulsions were prepared by blending TO with OA to stabilize the oil phase. The morphological, structural, and thermal properties of the microcapsules were comprehensively analyzed and comparatively evaluated. The combination of thermal regulation, bioactivity and biodegradable shell materials along with the stimuli-responsive behavior makes these microcapsules promising candidates for active food packaging, transportation of temperature-sensitive perishable products, biomedical dressings, protective coatings, cosmetic and personal care products, pharmaceutical and biomedical delivery systems, agricultural carriers and functional textiles.

## 2. Materials and Methods

### 2.1. Materials

**Figure 2:**
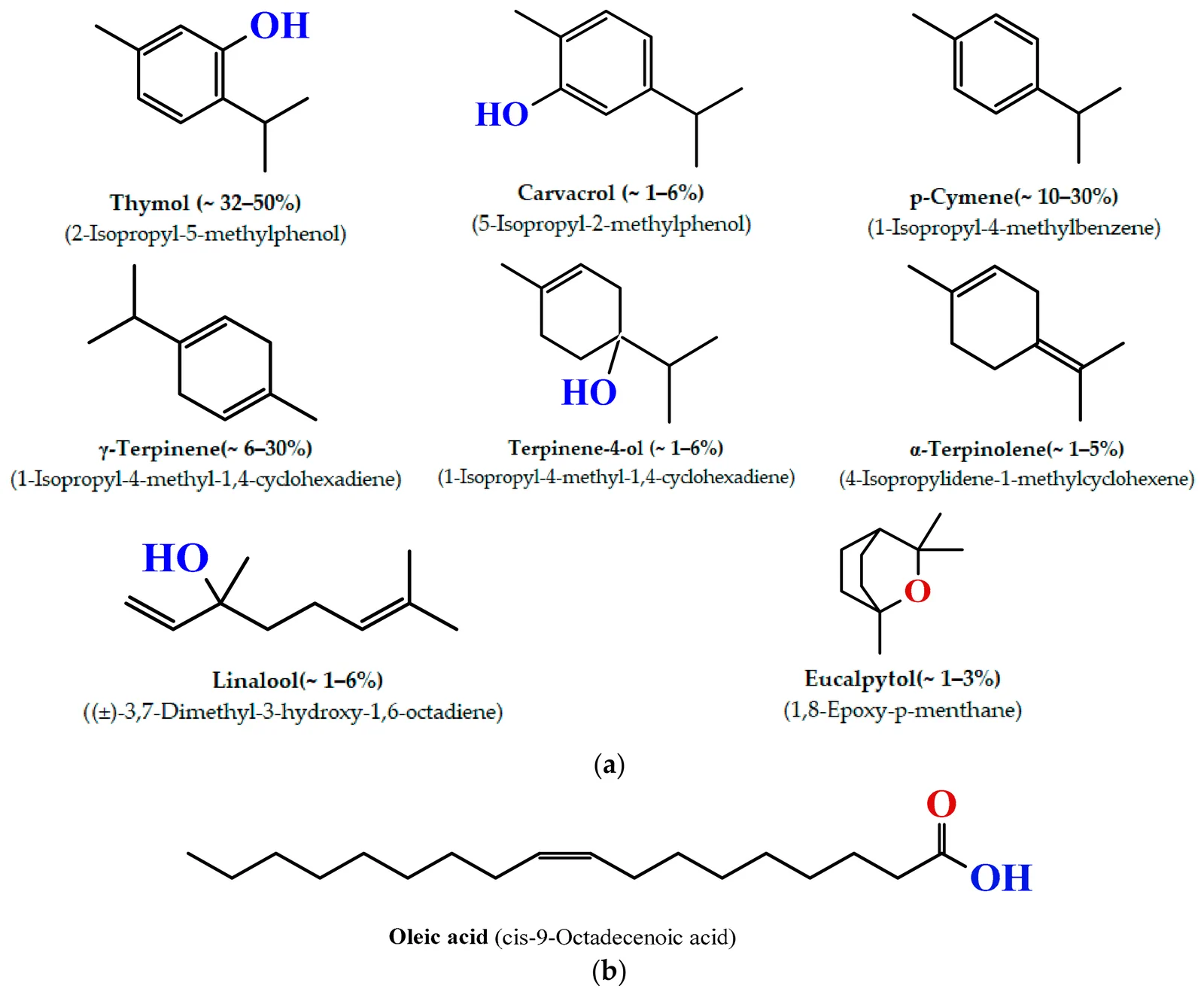
Skeletal structures of (**a**) the main components of thyme essential oil (adapted from Kowalczyk et al. [27]) and (**b**) oleic acid.

Alginate (Na[C_6_H_7_O_6_]_n_; sodium alginate; CAS No 9005-38-3; MW = 20–40 kDa, M/G = 1.56), poly(ethylene glycol)s (PEGs; [H(OCH_2_CH_2_)_n_OH]; CAS No. 25322-68-3), including PEG1000 (MW = 950–1050 Da; hydroxyl value = 107–118 mg KOH·g^−1^; d = 1.2 g·cm^−3^) and PEG1500 (MW = 1400–1600 Da; hydroxyl value = 70–80 mg KOH·g^−1^; d = 1.2 g·cm^−3^), oleic acid (CH_3_(CH_2_)_7_CH=CH(CH_2_)_7_COOH; cis–9–Octadecenoic acid; CAS:112-80-1; MW = 282.46 Da; d = 0.89 g·cm^−3^; HLB = 11.0; 90% purity), glacial acetic acid (analytical; CH_3_COOH), sodium chloride (NaCl), calcium chloride (CaCl_2_·2H_2_O), and hydrochloric acid (HCl; 37%) were all purchased from Merck KGaA (Darmstadt, Germany). Chitosan ([C_6_H_11_NO_4_]_n_; CAS No: 9012-76-4; MW= 190–310 kDa; 75–85% deacetylated) was supplied from ACROS Organics (Morris, NJ, USA). Thyme essential oil (Thyme oil; Thymus sp. Oleum; d = 0.917 g·cm^−3^; HLB = 9–9.5) was supplied commercially (Arifoğlu Spice and Food Industry & Trade Co., Istanbul, Türkiye). The skeletal structures of the shell materials, namely alginate, chitosan, and PEG, are shown in Figure 1. Figure 2 shows the structures of the main components of thyme essential oil and oleic acid, both of which form a core mixture.

### 2.2. Methods

#### 2.2.1. Preparation of Shell and Core Mixtures and Bath Solutions

A 1.75 wt% alginate solution [AL(aq)] was prepared by dissolving 1.75 g of sodium alginate in 100.0 mL of distilled water at 70 °C. This was performed using an Ultra-Turrax homogenizer (Vortex Genius 3, IKA-Werke GmbH & Co. KG (Staufen, Germany)), which was set to 21,000 rpm for 20 min. A 2.0 wt% chitosan solution [CH(aq)] was prepared by dissolving 2.0 g of chitosan in 100.0 mL of a 1.0% (*v*/*v*) acetic acid solution. This was then stirred continuously at 3800 rpm using the same Ultra-Turrax homogenizer at 75 °C until a clear homogeneous solution was obtained. A 0.1 M HCl (aq) solution was prepared from 37% HCl(aq) to adjust the pH.

The shell mixtures were produced by adding PEG1000 and/or PEG1500 to the alginate solution at different mass ratios. The reason for including these components in the shell mixture is twofold. The first reason is to enhance the stability of the resulting microcapsules, as shown by Li et al. [50], and the second reason is to provide microcapsules with heat storage and release capabilities by utilizing the PCM properties of PEGs. Initially, PEG1000 and PEG1500 were melted in a 60 °C water bath at mass ratios of 1:0, 0:1, and 1:1. The molten PEGs were then added to 25.0 mL of the 1.75 wt% AL(aq) solution at the same temperature and stirred at 1500 rpm for 30 min to achieve homogeneity. The core nanoemulsions were prepared by blending thyme oil (TO) with oleic acid (OA), which served as a surfactant, at a mass ratio of 1.1:2.5. The mixture was subjected to ultrasonication using a Selecta Optic Ivymen System Ultrasonic Homogenizer (CY-500 model, J.P. Selecta, Barcelona, Spain) at a frequency of 24 kHz, power of 400 W, with a cycle of 0.5, and amplitude of 70–80% for 10 min at room temperature.

To improve the alginate concentration gradient throughout the thickness of the shell and ensure homogeneous hardening, Bath 1 was prepared with 50.0 mL of 0.30 M CaCl_2_(aq) as the primary cross-linking agent and 12.5 mL of 0.20 M NaCl(aq) as the secondary cross-linking agent, in accordance with the literature [51,52], and adjusted to pH 6.40. Bath 2 was prepared by mixing 30.0 mL of 2.0 wt% chitosan (aq), 30.0 mL of 0.30 M CaCl_2_(aq), and 10.0 g of PEG1000 and/or PEG1500.

Table 1 shows the composition, pH, and conductivity values of the ALPEG microcapsule formulations. The pH values and electrical conductivities of the shell and core mixtures were measured at 25 °C using a Mettler Toledo S80 model Multi Conductometer (Mettler-Toledo, LLC, Columbus, OH, USA). The conductometer was calibrated with a standard solution (84 µS·cm^−1^), and the cell constant was set to ≤0.1. Table 2 shows dynamic viscosities of the shell and core solutions used in the electrospraying process. The dynamic viscosities of the solutions were measured using an SNB-1 model Stepless Speed Regulation Rotary Display Viscometer (Ningbo Hinotek Instrument Co., Ningbo, China) at a shear rate (R) of 60 rpm and 25 °C. The conductivity of Bath 1 was measured as 3.18 mS. The dynamic viscosities of Bath 1 and Bath 2 were measured as 0.05 Pa.s and 0.06 Pa.s, respectively.

#### 2.2.2. Microcapsule Production

Microcapsule production involves a sequential three-step process: (i) coaxial electrospraying of shell–core microcapsules; (ii) ionotropic gelation of the alginate–PEG matrix in a Ca^2+^ bath for shell stabilization; and (iii) polyelectrolyte complexation of chitosan–PEG over the primary shell. Electrospraying was performed using a coaxial electrospinning device (Yflow Co., Campanillas, Málaga, Spain) equipped with two coaxial needles with inner diameters of 0.6 and 1.4 mm, a double-polarized system (±30 kV), a grounded flat collector, and a Taylor cone visualization system. The electrospraying parameters, as detailed in Table 2, facilitated the formation of a Taylor cone anchored at the nozzle tip, thereby enabling the stable production of microcapsules. The collector was positioned 15 cm from the spinneret, and the electrospraying process was continued at 25 °C for 15 min without any interruption for each microcapsule type. During electrospraying, the TO-OA core emulsion was pumped through the inner needle, and the alginate–PEG shell mixture was pumped from the outer needle of the coaxial device. To produce core-free (hollow) microcapsules, the inner injector was operated at the same flow rate while being left empty, thereby introducing air into the capsules during electrospraying.

The shell–core capsules were directly deposited into Bath 1, which was placed on the collector. The produced microcapsules were collected from Bath 1 every 15 min to ensure consistent hardening while the electrospraying process proceeded. Upon contact with the cross-linking solution, the alginate chains in the shell matrix underwent ionic cross-linking with Ca^2+^ and Na^+^ ions, leading to the solidification of the viscous microcapsule walls, as mentioned previously by Loquercio et al. [53], who demonstrated the effective ionotropic gelation of alginate in the presence of calcium ions for nanoparticle formation, and by LeRoux et al. [52], who reported that both sodium and calcium ions significantly influence the mechanical properties of alginate gels. In the subsequent step, the capsules were transferred to Bath 2 and incubated for 10 min. During this stage, electrostatic interactions occurred between the carboxyl (–COO^−^) groups of alginates and the amino (–NH_2_) groups of chitosan, driving the formation of a polyelectrolyte complex [50,54]. Concurrently, additional hardening of the microcapsules occurred via Ca^+2^-mediated cross-linking of alginate and chitosan chains [55,56,57]. Following the secondary shell hardening process, the microcapsules were retrieved from Bath 2, washed with distilled water, and dried in a sealed glass plate. To verify the reproducibility of the shell–core microcapsules, the entire fabrication process was repeated four times under the same conditions.

**Figure 3:**
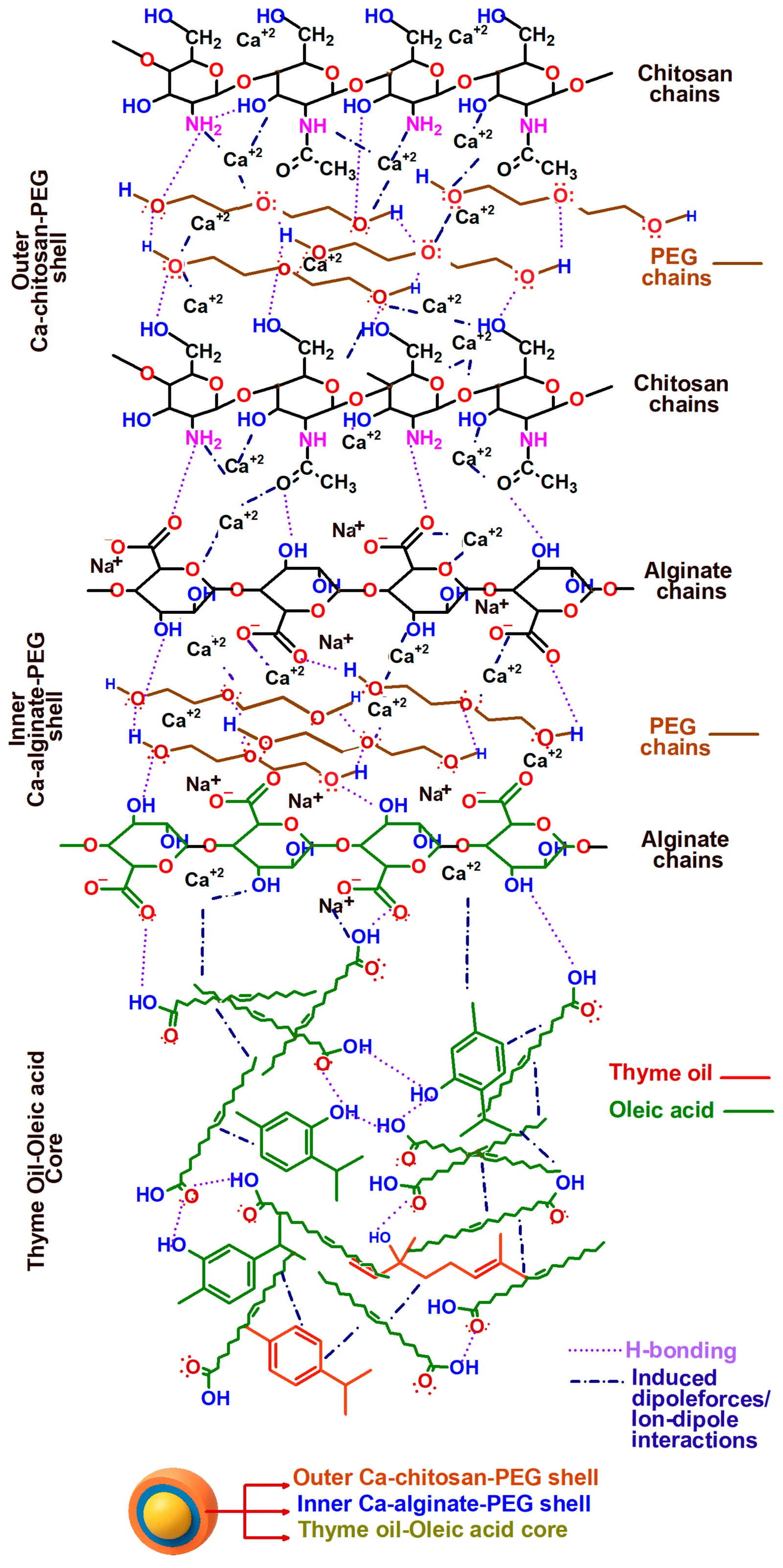
Proposed skeletal structure of the ALPEG microcapsules.

The proposed skeletal structure of the shell–core microcapsules is shown in Figure 3. A total of six different microcapsule types were produced to examine the effects of PEG type and the presence of the TO-OA core on microcapsule structure and properties, using the ALPEG prefix. In addition, the AL-Control sample was prepared, which contained only alginate and chitosan in the shell with no core (Table 1 and Table 2).

#### 2.2.3. Characterization of the Microcapsules

##### Structural Characterization of Microcapsules

Scanning Electron Microscope (SEM) images of the microcapsules were obtained using a Zeiss EVO LS 10 model SEM (Carl Zeiss Microscopy GmbH, Oberkochen, Germany) at 15 kV. To prepare the SEM samples, dried microcapsules were placed on standard mounts (15 mm × 2 mm) under vacuum and coated with a 1–2 nm thick conductive layer of gold to prevent charging during the imaging process.

The Fourier transform infrared (FTIR) spectra of the ALPEG microcapsules were recorded between 4000 and 650 cm^−1^ at a resolution of 4 cm^−1^ using a PerkinElmer Spectrum 100 FTIR Spectrometer (PerkinElmer Life and Analytical Sciences, Inc., Shelton, WA, USA) equipped with a universal attenuated total reflection (ATR) accessory.

##### Thermal Characterization of Microcapsules

Thermogravimetric (TG) analyses were carried out from 25.0 °C to 600.0 °C at 10.0 °C·min^−1^ heating rate under a dry nitrogen atmosphere purged at 150.0 mL·min^−1^ by a SEIKO EXSTAR 6200 Model TG/DTA instrument (Seiko Instrument Inc., Chiba, Japan). Differential scanning calorimetry (DSC) analyses were performed to examine and compare the thermal properties of the ALPEG microcapsules and their PEG and oleic acid components using a PerkinElmer DSC 4000 instrument (PerkinElmer Life and Analytical Sciences, Inc., Shelton, WA, USA) with an accuracy of ±0.001. Nitrogen flux (20 mL·min^−1^) was used as the purge gas for the furnace. Temperature scans were performed on the samples as successive heating–cooling cycles at 10.0 °C·min^−1^ between −50.0 °C and 80.0 °C, including the phase change temperature intervals of the PEGs used.

##### pH-Dependent Swelling Degree and Water Solubility of Microcapsules

The pH-dependent swelling degrees of the microcapsules were examined using buffer solutions with pH values of 3.0 and 7.0. The dried microcapsule samples were weighed (*m_ini_*) in Petri dishes. The buffer solution (2.0 mL) was added to the test sample and kept at 20 °C for 30 min. At the end of the period, the excess solution on the sample was removed with filter paper, and the wet sample was weighed (*m_wet_*). For continued swelling analysis, the same volume of buffer solution was added to the test samples, and wet mass measurements were repeated. The swelling experiments were conducted for a total duration of 60 min, with mass measurements at 30 and 60 min. The swelling (S%) was calculated using Equation (1).

(1) $$Swelling\;\left(S\%\right)=\left[\left(m_{wet}-m_{ini}\right)/m_{ini}\right]\times 100\%$$

Following the final wet weighing in the swelling test, the specimens were kept in a buffer solution for 24 h. Each specimen was removed from the solution, placed in a Petri dish, and dried in an oven at 40 °C for 2 h, and the final dry mass was measured (*m_final_*). The solubility in the water phase was calculated using Equation (2):

(2) $$Water\;Solubility\;\left(WS\%\right)=\left[\left(m_{ini}-m_{final}\right)/m_{ini}\right]\times 100\%$$

### 2.3. Dynamic Light Scattering (DLS) Experiments

In addition to investigating the pH-dependent swelling behavior of ALPEG microcapsules at ambient temperature, dynamic light scattering (DLS) measurements were performed using their prepared aqueous suspensions on a DLS instrument (Nano ZS90, Malvern Pananalytical Instruments Ltd., Malvern, WR, UK) to comparatively evaluate temperature-dependent hydrodynamic behavior of the ALPEGs’ dispersed particles. Before analysis, 3.0–5.0 mg of dry ALPEG microcapsules were diluted with 5.0 mL of distilled water in a clean glass vial. After adding 0.1 mL of ethanol (2% *v*/*v*), the prepared dispersions were first homogenized by shaking in a vortex for 30 s, followed by sonication for a further 30–60 s using an ultrasonic device at low power and then filtered through a 0.45 μm syringe filter to remove any possible aggregates to obtain a monodisperse suspension suitable for DLS measurements. The resulting suspensions were filled into a DLS cuvette without allowing bubble formation. The test specimens were initially kept at room temperature (20.0 °C) for 5 min and then subjected to the DLS thermal cycle procedure, which consisted of heating and subsequent cooling cycles from 20.0 °C to 55.0 °C and 55.0 °C to 20.0 °C at 5.0 °C·min^−1^, with pauses of two minutes at every 5.0 °C increment or decrement to measure temperature-dependent hydrodynamic diameter (Z_ave_) and polydispersity index (PDI) variations. The tests were performed in triplicate for each type of sample [44].

### 2.4. Total Phenolic Content and Free Radical Scavenging Capacity Determination

The following methods were conducted to assess the antioxidant activity of the ALPEG microcapsules spectrophotometrically in the visible range using Kasuaki IL-592-LC-BI model UV–visible spectrophotometer (Kasuaki Inc., Wuxi, China). The total phenolic content (TPC) of the samples was determined using the Folin–Ciocalteu method with some modifications [58]. Each microcapsule sample (30.0 ± 0.1 mg) was blended with 10.0 mL of pure water in a beaker at 45.0 °C for 60 min at 500 rpm. The liquid phase was separated via centrifugation for 10 min at 3000 rpm. The liquid phase (1.0 mL) was combined with 5.0 mL of 0.2 M Folin–Ciocalteu reagent at room temperature. Subsequently, 4.0 mL of 7.5% (*v*/*v*) Na_2_CO_3_(aq) solution was added, and the solution was thoroughly shaken. The samples were then stored in the dark at room temperature for 60 min. The absorbance values of the extracts obtained from the microcapsule samples were measured at 760 nm. The concentration of total phenolic compounds in the samples was determined in milligrams of gallic acid equivalent (GAE) per gram of microcapsule (mg GAE/g microcapsule) according to Equation (3), where (R^2^ = 0.9818, x = GAE in mg).

*Abs*_760_= 0.005 *x* − 0.0073 (3)

DPPH (2,2-diphenyl-1-picrylhydrazyl; M = 394.32 g·mol^−1^) free radical scavenging test was carried out to measure the antioxidant capacities of the ALPEG microcapsules as described by Mehdizadeh et al. [59] based on the scavenging of DPPH free radicals by antioxidants due to a redox reaction causing discoloration of the molecule. A total of 10.0 mg of each sample was mixed with 10.0 mL of ethyl alcohol, shaken well for 10 min, and then centrifuged at 1000 rpm. The supernatant (1.5 mL) was mixed with 1.5 mL of 0.06 mM DPPH (ethanol) solution and kept for 30 min in the dark. The DPPH free radical scavenging activity was determined using Equation (4):

(4) $$DPPH\;scavenging\;activity\;\%=\left[\frac{Abs_{DPPH}-Abs_{Sample}}{Abs_{DPPH}}\right]\times 100$$

*Abs_DPPH_* refers to the absorbance of a 0.06 mM DPPH(ethanol), while *Abs_Sample_* denotes the absorbance of the sample extracts measured at 517 nm.

## 3. Results and Discussion

### 3.1. Structural Characterizations of Microcapsules

#### 3.1.1. The Scanning Electron Microscope (SEM) Images of the Microcapsules

**Figure 4:**
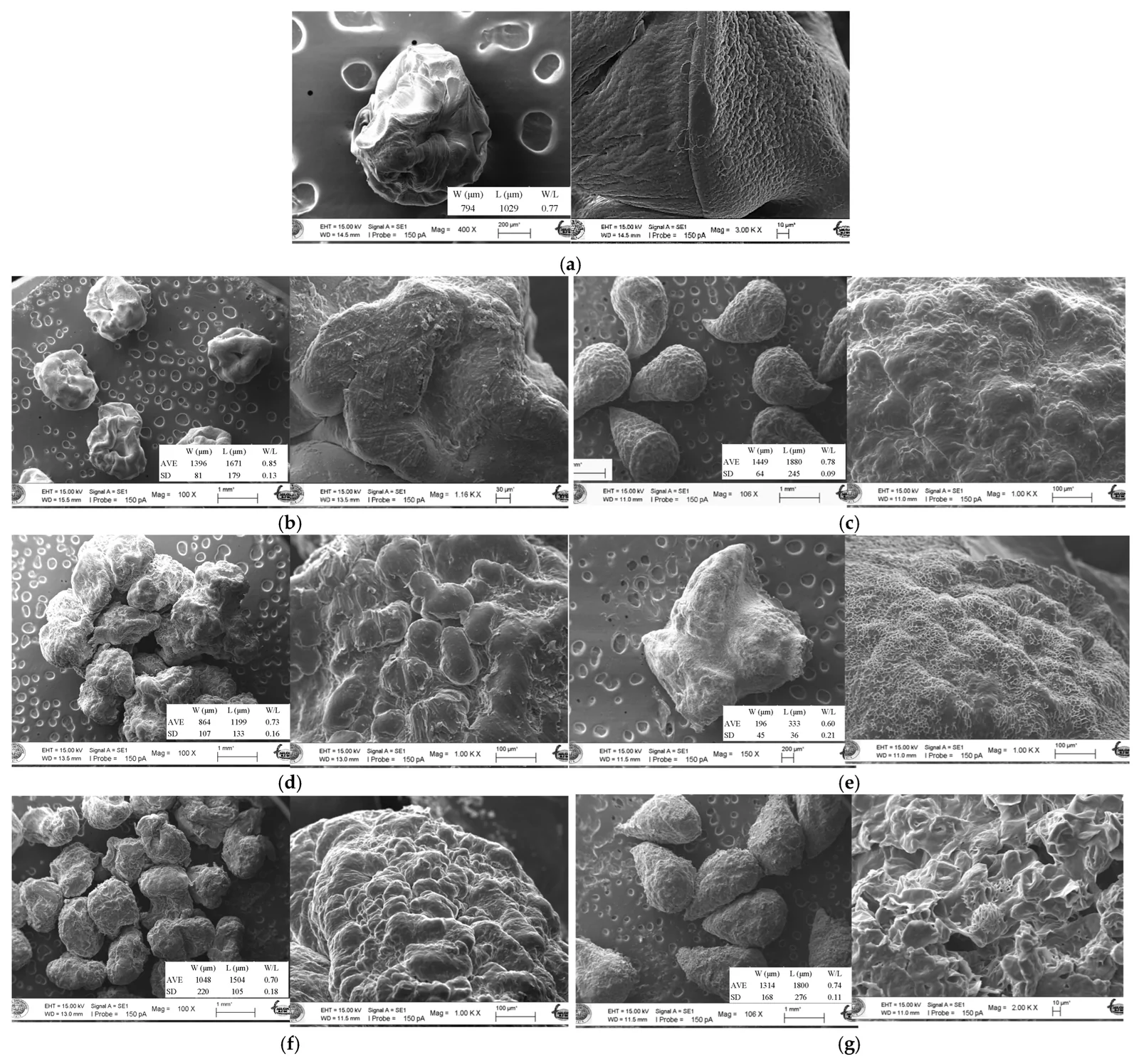
The SEM images of (**a**) AL-Control (×400–3000); (**b**) ALPEG-3 (×100–1160); (**c**) ALPEG-10 (×106–1000); (**d**) ALPEG-5 (×100–1000); (**e**) ALPEG-11 (×150–3000); (**f**) ALPEG-6 (×100–1000); (**g**) ALPEG-9 (×106–2000).

Figure 4a–g display the SEM images, shapes, sizes and surface morphologies of AL-Control and ALPEG microcapsules, characterized by the distinct microcapsule formations. Their sizes were measured in the width (W) and length (L) directions from their SEM images using Digimizer Image Analysis Software (version 4.6.1, MedCalc Software Ltd., Ostend, Belgium). The average (AVE) and standard deviation (SD) of the sizes of ALPEG-3, ALPEG-10, ALPEG-5, ALPEG-6 and ALPEG-9 were calculated from five capsule measurements. AL-Control size was measured from a single capsule and the values given for ALPEG-11 was obtained from three capsule measurements. Width-to-length (W/L) ratios of microcapsules were also calculated.

The AL-Control microcapsule exhibits a continuous and smooth surface with a slightly wrinkled texture, and has a non-spherical shape (794 µm *×* 1029 µm) with a width-to-length (W/L) ratio of 0.77. This morphology aligns with an alginate–chitosan double-layer structure cross-linked with Ca^2+^ and Na^+^ ions, as previously described by Chandy et al. [60] and Diana et al. [61] in similar polyelectrolyte microcapsule systems. Incorporating PEG1000 and/or PEG1500 into the shell structure resulted in larger capsule sizes and a bulkier surface morphology across all ALPEG samples. Additionally, microcapsules containing the TO-OA mixture in their cores showed reduced sphericity compared to their core-free counterparts as shown in their SEM images. For example, ALPEG-3 microcapsule sizes lie in a range of 1396 ± 81 µm *×* 1671 ± 179 µm with a W/L ratio of 0.85 ± 0.13, while ALPEG-10 microcapsule size range is 1449 ± 64 µm *×* 1880 ± 245 µm, with a W/L ratio of 0.78 ± 0.09. In the case of ALPEG-11 microcapsules, which contain PEG1500 in the shell, the observed capsule agglomeration and rougher surface morphology are consistent with findings by Chandy et al. [60], who noted that high molecular weight PEGs tend to alter the overall shape and size of the capsules and induce roughness due to their larger chain size. SEM images of ALPEG-3, ALPEG-5, and ALPEG-6, in the absence of TO-OA, reveal some localized inward depressions on the shell, attributed to the hollow microcapsule structure, similar to that of AL-Control. In contrast, SEM images of ALPEG-10, ALPEG-11, and ALPEG-9, which include the TO-OA core, imply a more compact and solid body with no apparent collapses. In summary, SEM analyses of AL-Control and ALPEG microcapsules confirm the successful fabrication of capsules with structural integrity through the combined processes of electrospraying, ionotropic gelation, and polyelectrolyte complexation. Comparative evaluation of the SEM images reveals that capsule size, shape, and surface morphology are influenced by both the incorporation of PEG1000 and/or PEG1500 within the shell matrix and the presence of the thyme oil–oleic acid (TO-OA) mixture in the core.

#### 3.1.2. FTIR Characterization of the Microcapsules

**Figure 5:**
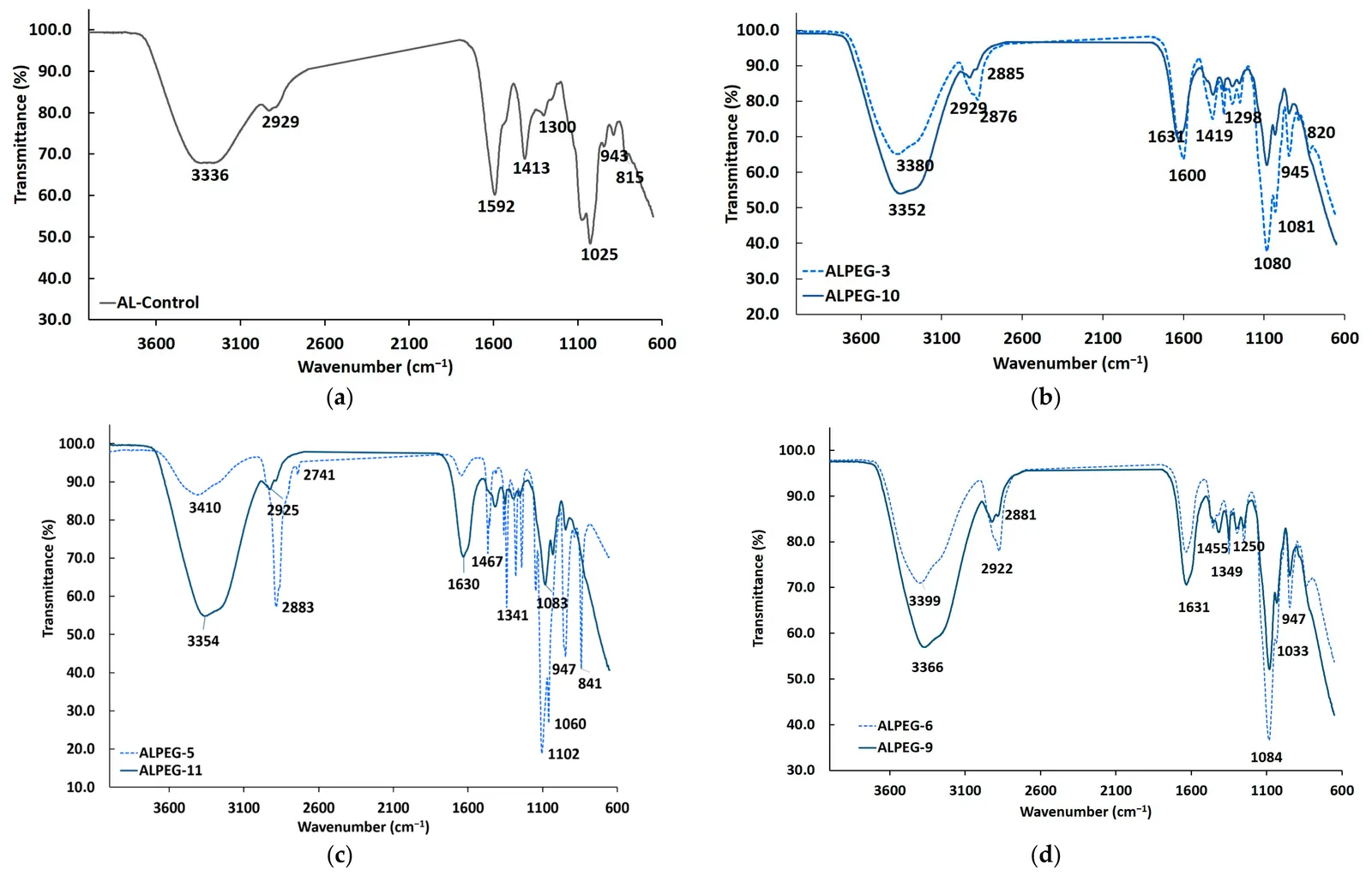
FTIR spectra of: (**a**) AL-Control; (**b**) ALPEG-3 and ALPEG-10; (**c**) ALPEG-5 and ALPEG-11; (**d**) ALPEG-6 and ALPEG-9 between 4000 and 600 cm^−1^.

Figure 5a–d illustrate the FTIR spectra of the AL-Control and ALPEG microcapsules. The FTIR spectra of pure Na-alginate and chitosan are presented and discussed in Supplementary S1. The FTIR analyses of PEG1000, PEG1500, thyme oil, and oleic acid are provided in Supplementary S2 and S3.

The FTIR spectrum of AL-Control (Figure 5a) exhibits the characteristic bands of Na-alginate and chitosan (Supplementary S1). The broad band at 3336 cm^−1^ corresponds to O–H and N–H stretching vibrations, indicating the presence of hydroxyl (–OH) and amine (–NH) groups within chitosan and alginate, as well as strong hydrogen bonding interactions between them [19], which contribute significantly to the structural integrity of the capsule shell. The presence of alginate is confirmed by the bands at 1592 cm^−1^ and 1413 cm^−1^, which are attributed to the symmetric and asymmetric stretching vibrations of the carboxylate (–COO^−^) groups in alginate, respectively [60]. The band at 1300 cm^−1^ is associated with –CH_2_ vibrations, while the 1025 cm^−1^ band corresponds to C–O and C–O–C stretching of glycosidic linkages. Bands at 943, 888, and 815 cm^−1^ represent skeletal vibrations of the polysaccharide backbone, confirming preservation of the alginate–chitosan structure. Overall, FTIR analysis verifies the formation of a stable alginate–chitosan polymeric network, consistent with the continuous and even surface morphology observed in SEM images (Figure 4a).

Incorporation of PEG into the alginate–chitosan matrix induced notable shifts across various FTIR regions (Figure 5b–d). The O–H/N–H stretching band shifted from 3336 cm^−1^ (AL-Control) to 3380–3410 cm^−1^ in the IR spectra of ALPEG-3, ALPEG-5, and ALPEG-6, indicating increased hydrogen bonding interactions caused by the incorporation of PEG1000 and/or PEG1500 into the alginate–chitosan matrix [62,63,64,65]. Minor shifts in the C–H stretching region (from 2929 to 2876–2884 cm^−1^) and upward shifts in the asymmetric and symmetric –COO^−^ bands (from 1592 to 1600–1645 cm^−1^ and from 1413 to 1416–1467 cm^−1^) further confirm structural conformational and electrostatic rearrangements in the polymer network upon PEG incorporation, consistent with previous reports on PEG-modified polysaccharide systems [66,67,68,69]. Further changes were observed in the 1300–1000 cm^−1^ region, where the emergence and intensification of bands attributed to C–O–C and C–O vibrations confirmed the presence of PEG chains within the matrix [19,66,70]. In the fingerprint region (950–800 cm^−1^), small but consistent shifts further indicated subtle conformational rearrangements of the polysaccharide backbone. Specifically, the 943 cm^−1^ band of AL-Control shifted to 945–947 cm^−1^ in ALPEG samples, and the 815–888 cm^−1^ region exhibited band broadening and partial splitting, consistent with the structural rearrangement reported for modified alginate–chitosan systems [60,66]. These spectral changes support the interaction between PEG and the alginate–chitosan matrix and the improvement in integration within the shell network, and are consistent with SEM observations regarding altered surface morphologies and shell architectures.

Encapsulation of the TO-OA core within ALPEG-9, ALPEG-10, and ALPEG-11 produced more pronounced shifts in the O–H/N–H region (up to 3352–3366 cm^−1^), indicating strengthened hydrogen bonding between TO-OA hydroxyl/carboxyl groups and the alginate–PEG–chitosan matrix (Figure 5b–d) [71]. Characteristic thyme oil bands in the 1464–1370 cm^−1^ range shifted or overlapped with alginate–PEG–chitosan matrix signals, and the development of new bands at 1349–1350 cm^−1^ confirms successful TO-OA core incorporation within the shell. The fingerprint region (1299–1000 cm^−1^) exhibited stronger and sharper C–O and C–O–C stretching bands, particularly at 1299–1251 and 1083–1033 cm^−1^, compared to PEG-only samples, suggesting enhanced shell–core interactions and possible esterification of the TO-OA core [70]. Shifts at 947–945 cm^−1^ further indicate polysaccharide backbone rearrangements and phenolic contributions from thyme oil [38,64]. Consistent with previous reports on thyme oil–polymer systems [39,47,60,72], the IR spectral modifications observed in the oil-loaded samples (ALPEG-10, ALPEG-11, and ALPEG-9) indicate successful incorporation of PEG into the shell matrix and effective encapsulation of TO-OA within the alginate–PEG–chitosan shell, evidenced by characteristic features of both the shell and core components [73]. The FTIR findings are consistent with SEM observations, suggesting that PEG incorporation and TO-OA encapsulation contribute to the physicochemical and structural properties of the ALPEG microcapsules.

### 3.2. Thermal Analysis Results of Microcapsules

#### 3.2.1. Thermogravimetric (TG/DTG) Analysis of Microcapsules

**Figure 6:**
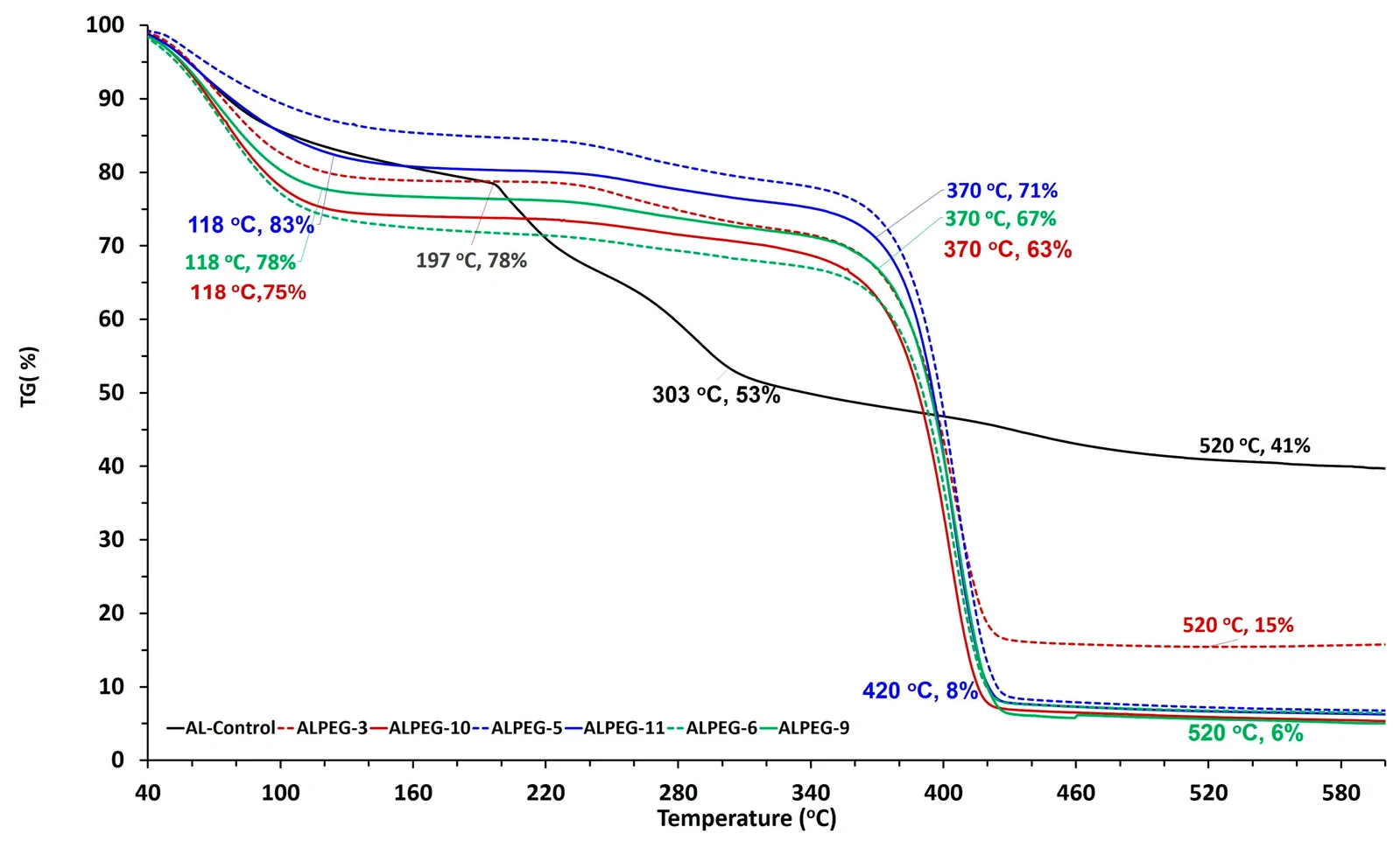
TGA curves of ALPEG microcapsules.

Figure 6 shows the mass loss % curves of the microcapsules in thermogravimetric (TG) analyses in the range of 25 °C to 600 °C, and Table 3 summarizes the corresponding TG data.

Compared with ALPEG samples, AL-Control exhibits a markedly different thermal degradation profile. For the AL-Control sample, the 15% mass loss observed from 20.0 °C to 118.0 °C is associated with the evaporation of free moisture and bound water retained within the alginate–chitosan network structure. The AL-Control underwent more rapid thermal decomposition than other microcapsules, which occurred between 197.0 °C (22% mass loss) and 303.0 °C (47% mass loss), due to the breaking of glycosidic bonds, hydroxyl, carboxyl, and carbonyl groups of alginates [45,74], and due to the fracturing of saccharide rings and functional groups, such as CO, OH and NH_2_, and partial depolymerization of the chitosan chains [75]. Beyond this point, the mass loss of AL-Control became slower, and the mass percent reduced to 48% at 370.0 °C, ultimately reaching approximately 41% at 520.0 °C. The remaining residue is attributed to thermally stable carbonaceous structures derived from alginate and chitosan, together with inorganic Na and Ca salts originating from the cross-linking process [76].

All types of ALPEG microcapsules exhibited a three-step thermal decomposition curve in their thermograms. The dehydration of the microcapsules continued up to 118.0 °C, associated with the evaporation of water molecules within their structures, leading to mass losses ranging between 12 and 26%. Unlike AL-Control, ALPEG microcapsules exhibited reduced mass loss, by 26–37%, and delayed degradation in the 200.0–370.0 °C range, indicating enhanced thermal stability due to hydrogen bonding interactions between PEG chains and alginate–chitosan networks. The presence of PEG effectively restricts polysaccharide chain mobility and retards backbone scission, consistent with previous reports on PEG-modified biopolymer systems [75]. ALPEG-5 and ALPEG-11 samples, containing PEG1500, notably showed higher mass retention in this region, reflecting the increased thermal resistance associated with longer PEG chain lengths [77]. The thermal decomposition of all ALPEG samples accelerated between 370.0 °C and 420.0 °C, and their mass losses reached 85–90%, mostly governed by the evolution of PEG oligomers and pyrolytic reactions [78]. At higher temperatures (420.0–520.0 °C), ALPEG samples displayed a much lower residual mass than AL-Control, by around only 6–7%, which is mainly attributed to the reduced amount of alginate–chitosan char and inorganic salts remaining after the extensive thermal decomposition of PEG components around 450.0 °C [77].

The ALPEG-10 and ALPEG-11 microcapsules, which encapsulate TO-OA in their cores, exhibited slightly higher mass losses (25% and 17%) at 118.0 °C compared to their ALPEG-3 and ALPEG-5 counterparts (20% and 12%), possessing identical shell compositions but lacking a core, likely due to the volatilization of TO-OA core diffused through the shell [37,79,80]. Furthermore, as shown from DSC analysis given in Supplementary Figure S5, thyme oil (TO) evaporates between 109 °C and 155.8 °C, peaking at 153.2 °C. Based on this thermal behavior, TGA mass losses were also evaluated at 156 °C, yielding 26% for ALPEG-10 and 19% for ALPEG-11. Conversely, the core-free capsules (ALPEG-3 and ALPEG-5) lost only 21% and 14% of their mass at this temperature. This consistent 5% increase in mass loss for ALPEG-10 and ALPEG-11 is an important indication of successful encapsulation of thyme oil in the core in accordance with the literature [80].

#### 3.2.2. Differential Scanning Calorimeter (DSC) Results of ALPEG Microcapsules

**Figure 7:**
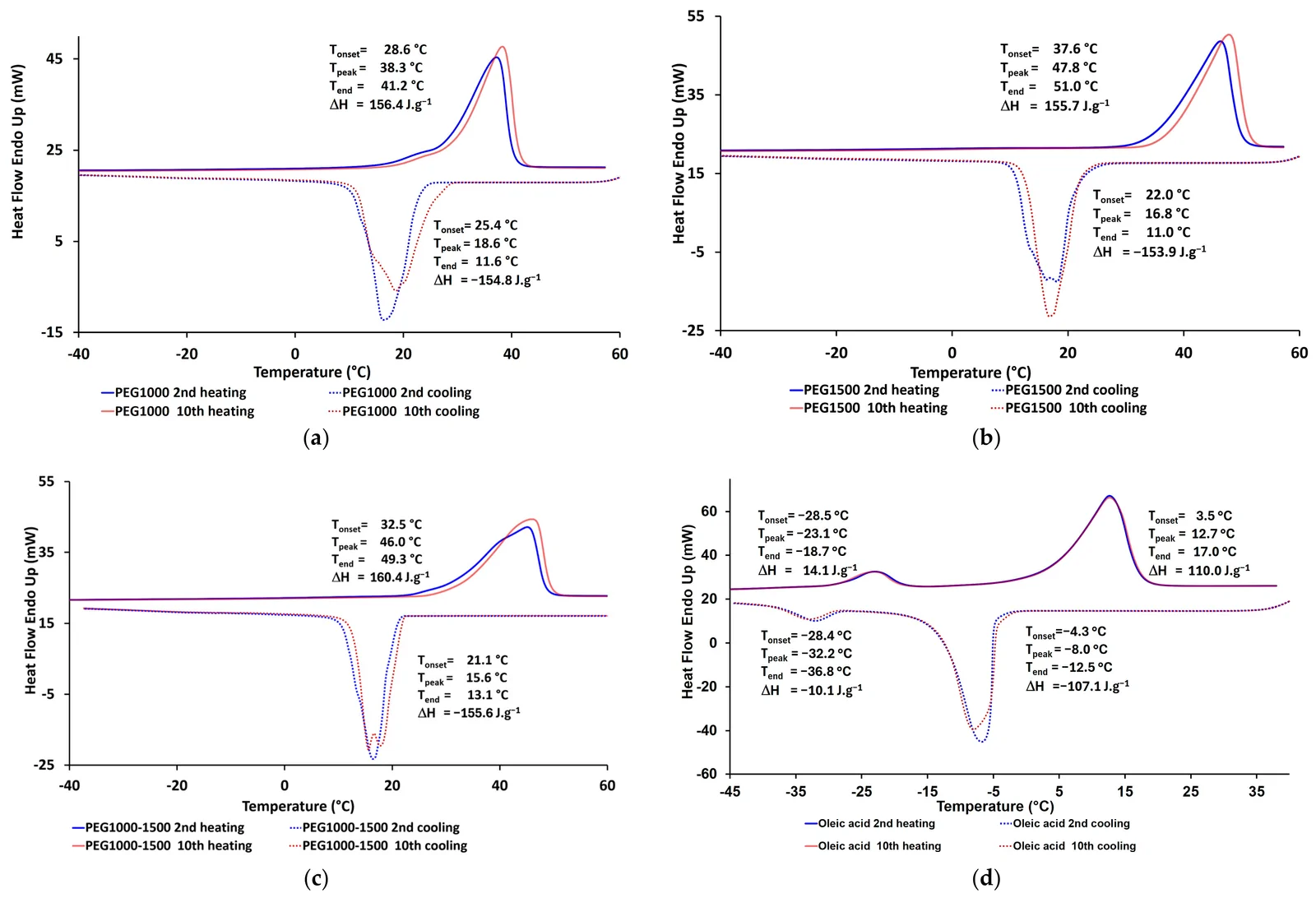
DSC graphs of PEGs used in the shell mixtures and oleic acid used as a surfactant in the core mixtures for 2nd and 10th heating–cooling cycles at 10.0 °C·min^−1^: (**a**) PEG1000; (**b**) PEG1500; (**c**) 50/50 blend of PEG1000/PEG1500; (**d**) oleic acid.

**Figure 8:**
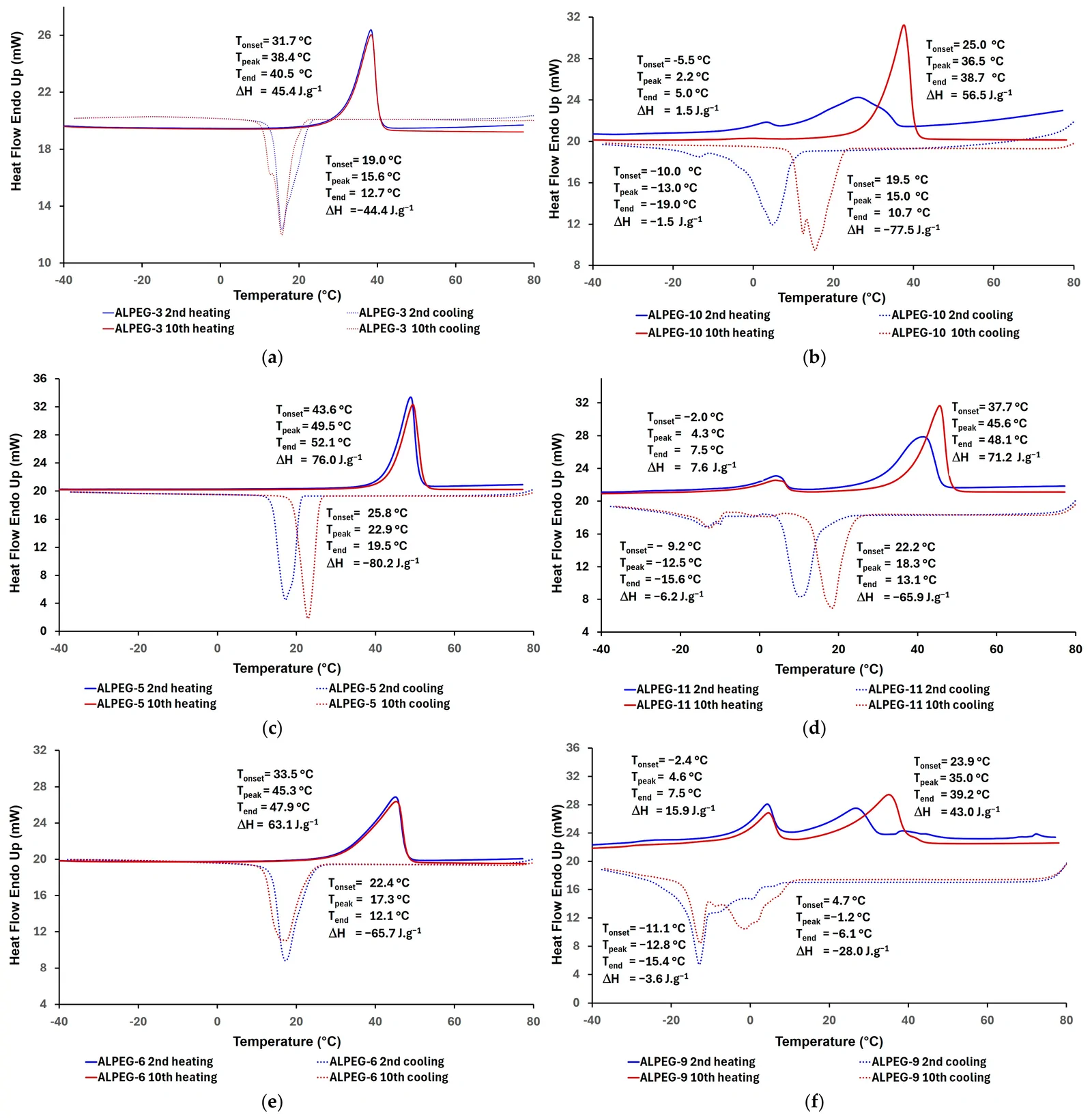
DSC 2nd and 10th heating–cooling cycles of (**a**) ALPEG-3; (**b**) ALPEG-10; (**c**) ALPEG-5; (**d**) ALPEG-11; (**e**) ALPEG-6 and (**f**) ALPEG-9 microcapsules at 10.0 °C·min^−1^.

The DSC thermograms of PEGs incorporated into the shell of the ALPEG microcapsules and of oleic acid are presented in Figure 7a–c. The DSC graph of PEG1000 exhibits a peak phase transition temperature of 38.3 °C with a melting enthalpy of 156.4 J·g^−1^, while that of PEG1500 displays a peak phase transition temperature of 47.8 °C and a melting enthalpy of 155.7 J·g^−1^, respectively, confirming the characteristic phase change behavior of these PCMs [40]. The peak phase transition temperature of 46.0 °C and the melting enthalpy of 160.4 J·g^−1^ observed for the 50/50 PEG1000/PEG1500 PCM blend indicate a phase change behavior intermediate between those of neat PEG1000 and PEG1500. DSC analyses were performed in triplicate for each ALPEG microcapsule type, with test specimens selected from different production batches. Table 4 presents the comparative DSC results of the ALPEG microcapsules obtained from the 10th heating–cooling cycle, including three individual measurements for each sample and their corresponding mean values with standard deviations. Their representative DSC graphs are shown in Figure 8a–f.

The DSC results clearly demonstrate that the phase change behavior of ALPEG microcapsules is governed by the type and molecular weight of the PEG incorporated into the shell. Upon encapsulation, all ALPEG samples showed broadened phase transition intervals, shifted onset and peak temperatures, and remarkable transition enthalpies directly related to the effective incorporation and retention of PEGs within the shell structure. The variations observed in the onset, peak, and end temperatures of the phase transitions among the three repeated measurements for each sample can be attributed to the hybrid nature of the alginate–PEG–chitosan shell matrix. During cooling cycles, phase transition temperature shifts toward lower values are associated with the freezing point depressions arising from the typical solidification behavior of the PEGs, as well as with the hybrid shell structure [81].

As seen from Table 4, the onset, peak, and end temperatures of ALPEG-10 in the 10th heating cycle were observed at 25.3 ± 7.4 °C, 37.6 ± 3.1 °C, and 40.9 ± 2.3 °C, respectively, closely matching the phase transition range of PEG1000 (T_onset_ = 28.6 °C; T_peak_= 38.3 °C; T_end_= 41.2 °C). Its mean enthalpy of 70.5 ± 12.5 J·g^−1^ corresponds to a thermal storage efficiency of 51.2 ± 9.1%, calculated relative to the expected enthalpy (ΔH_expected_ = 137.6 J·g^−1^) based on the PEG1000 content in the shell composition. For ALPEG-11, the onset, peak, and end temperatures were recorded at 37.4 ± 5.2 °C, 46.4 ± 3.6 °C, and 49.6 ± 3.1 °C, respectively, corresponding well to the phase transition interval of PEG1500 (T_onset_ = 37.6 °C; T_peak_ = 47.8 °C; T_end_ = 51.0 °C). The thermal storage efficiency of ALPEG-11 was calculated as 58.4 ± 8.3%, based on its mean enthalpy value of 80.0 ± 11.4 J·g^−1^ and the expected enthalpy (ΔH_expected_ = 137.0 J·g^−1^) estimated from the PEG1500 content in the shell composition. ALPEG-9 exhibited the onset, peak, and end temperatures of 31.7 ± 3.7 °C, 44.5 ± 2.9 °C, and 47.2 ± 2.8 °C, respectively, consistent with the thermal behavior of the 50/50 PEG1000/PEG1500 blend (T_onset_ = 32.5 °C; T_peak_ = 46.0 °C; T_end_ = 49.3 °C). Its mean enthalpy value of 75.8 ± 2.9 J·g^−1^ corresponds to the thermal storage efficiency of 53.7 ± 2.1%, based on the expected enthalpy (ΔH_expected_ = 137.0 J·g^−1^) of 50/50 PEG1000/PEG1500 content in the shell composition (Table 1 and Figure 7a–c). In addition, remarkably, the DSC curves of ALPEG-10, ALPEG-11, and ALPEG-9 reveal a subtle shoulder in the low-temperature region (around and below 0 °C) during heating and cooling cycles (Figure 8b,d,f). This feature suggests a minor contribution of the phase transition behavior of oleic acid in the core, indicating a limited but detectable influence of the TO-OA core on the overall thermal response of the microcapsules. The DSC results for core free ALPEG-3, ALPEG-5, and ALPEG-6 closely match those of their respective shell counterparts, in particular the highest enthalpy for 91.8 ± 18.6 J·g^−1^ at 48.5 ±1.9 °C with the thermal storage efficiency of 67.0 ± 13.6% for ALPEG-5, but show no additional low-temperature thermal transitions, consistent with the absence of the TO-OA core (Table 4 and Figure 8a,c,e).

### 3.3. pH-Dependent Swelling Degree and Water Solubility Results of ALPEG Microcapsules

The pH-dependent swelling degree (S%) of the ALPEG microcapsules in buffer solutions at pH = 3.0 and pH = 7.0 at 30 min and 60 min, and their water solubility (WS%) at 24 h are given in Table 5. Table 5 shows that all ALPEG microcapsules exhibited pronounced pH- and time-dependent swelling behavior, with substantially higher swelling degrees at pH 7.0 than at pH 3.0, irrespective of shell composition. This behavior can be attributed to the increased affinity of water molecules for the hydroxyl, carboxyl, and carbonyl groups of alginates, as well as the hydroxyl and amine groups of chitosan under neutral conditions, which promotes matrix hydration and expansion. In contrast, protonation under acidic conditions limits polymer chain mobility and restricts network swelling [82].

At pH 3.0, swelling increased gradually with time for all samples. Compared to AL-Control, PEG-containing microcapsules generally showed enhanced swelling, particularly ALPEG-3, which exhibited the highest swelling ratios at both time points (S% ratios of 77.3% and 168.6% at 30 and 60 min, respectively). In contrast, ALPEG-5 and ALPEG-11, containing PEG1500, displayed more limited swelling at acidic pH, suggesting a denser or less permeable shell structure associated with the longer PEG chains. At pH 7.0, swelling degrees increased dramatically with immersion time, reaching values of 36.6–196.8% at 30 min and 372.2–948.4% at 60 min. These results indicated that PEG incorporation significantly enhanced water uptake and matrix expansion, particularly under neutral conditions, while acidic pH limits swelling due to restricted polymer chain mobility [82].

Comparisons between microcapsule pairs with identical shell compositions but different cores (ALPEG-3 vs. ALPEG-10, ALPEG-5 vs. ALPEG-11, and ALPEG-6 vs. ALPEG-9) show that the presence of the TO-OA core generally led to relatively lower swelling percentages at both 30 and 60 min. This trend suggests that the hydrophobic nature of the TO-OA partially limited water penetration into the capsule structure, thereby reducing the extent of network expansion in core-loaded microcapsules. Similar effects of hydrophobic cores on swelling behavior have been reported in the literature [83,84].

As shown in Table 5, the water solubility (WS%) values of the microcapsules within 24 h were generally high under acidic conditions, reflecting the presence of chitosan in the capsule structure. For example, AL-Control exhibited a WS% of 86.2% at pH 3.0, which decreased to 74.9% at pH 7.0. Samples containing PEG1000 (ALPEG-3, ALPEG-10, ALPEG-6, and ALPEG-9) showed remarkably higher WS% values at pH 7.0, reaching 96.0–100.0%. This behavior is consistent with their elevated swelling degrees and reflects the hydrophilic nature of PEG-containing shells. On the other hand, ALPEG-5 and ALPEG-11, both containing only PEG1500, displayed lower solubility values at both pH levels, particularly at pH 7.0 (66.8% and 61.4%, respectively), suggesting a more cohesive and less readily dissolving network.

### 3.4. Dynamic Light Scattering (DLS) Measurements

The aim of DLS measurements was to evaluate the temperature-dependent swelling–deswelling behavior of particles dispersed in aqueous suspensions of each ALPEG type, not to measure the original capsule sizes. It was also aimed to determine and compare the differing temperature responses of the suspensions, prepared from different capsule types, during the applied thermal cycling process. In fact, initial particle sizes of each suspension, at the beginning of DLS measurements, varied greatly owing to their swelling characteristics since each suspension was held at 20 °C for 5 min before the test. DLS results revealed that dispersed particles in all prepared aqueous suspensions exhibited reversible, temperature-dependent hydrodynamic diameter (Z_ave_) variations during heating and cooling cycles [44] (see Table 6 and Supplementary S4).

The DLS measurements of AL-Control dispersion demonstrated reversible size changes during heating–cooling cycles, exhibiting an expansion–contraction behavior of up to 3.0–3.2-fold relative to its initial Z_ave_ value of 296 ± 34 nm at room temperature, attributable to the hydrogel-based swelling–deswelling nature of the alginate–chitosan matrix [45]. Compared to AL-Control, the dispersions of all ALPEGs exhibited larger initial Z_ave_ values in the range between 426 ± 85 nm and 898 ± 11 nm (T = 20.0 °C). ALPEG dispersions also displayed reversible temperature-dependent size variations as expansion upon heating and contraction upon cooling. However, the variations in the measured hydrodynamic sizes occurred within a relatively narrow range, say up to 896 ± 43 nm for ALPEG-3 (2.1-fold), 691 ± 28 nm for ALPEG-10 (1.5-fold), 1698 ± 53 nm for ALPEG-5 (2.2-fold), 879 ± 271 nm for ALPEG-11 (1.6-fold), 654 ± 8 nm for ALPEG-6 (1.1-fold), and 1124 ± 50 nm for ALPEG-9 (1.3-fold), upon heating. During the cooling cycles, the Z_ave_ values of the dispersions largely returned to their initial sizes. The mean particle sizes were reduced to 424 ± 60 nm for ALPEG-3 (2.1-fold), 621 ± 24 nm for ALPEG-10 (1.1-fold), 1313 ± 56 nm for ALPEG-5 (1.3-fold), 627 ± 13 nm for ALPEG-11 (1.4-fold), 526 ± 20 nm for ALPEG-6 (1.2-fold), and 893 ± 29 nm for ALPEG-9 (1.3-fold) upon cooling. The more restricted expansion and contraction observed in ALPEG-10 and ALPEG-11 dispersions compared to their counterparts ALPEG-3 and ALPEG-5 can be attributed to the presence of TO-OA in their core [85]. Nevertheless, they still exhibited significantly temperature-dependent swelling–deswelling behavior.

The polydispersity index (PDI) values of the prepared ALPEG suspensions are given in Supplementary S4. Typically, PDI values below 0.05 indicate a monodisperse system, whereas values above 0.7 correspond to a broad, polydisperse distribution [86]. The PDI values of AL-Control and ALPEG suspensions remained below 0.7 at room temperature (20.0 °C), confirming that the prepared dispersions are within the appropriate range for DLS measurements [87]. During heating up to 55.0 °C, PDI values indicated a shift towards a more heterogeneous dispersion for all samples. However, in ALPEG-10 and ALPEG-11, the temperature-induced changes in PDI values still remained around 0.7 or below; for example, the PDI value of ALPEG-10 increased from 0.361 ± 0.058 at 20 °C to 0.644 ± 0.040 at 50.0 °C, and PDI value of ALPEG-11 increased from 0.525 ± 0.114 at 20.0 °C to 0.656 ± 0.022 at 50.0 °C.

Overall, in DLS measurements, the observed two-directional size changes and PDI values of the suspensions are indicative of the temperature-dependent, transient behavior rather than permanent structural changes. These findings support that the temperature-induced hydrogel behaviors of ALPEG microcapsules in double-layer alginate–PEG–chitosan shell systems are promising for such a shell–core capsule designs intended to achieve sustained release characteristics [88,89,90,91].

### 3.5. Antioxidant Activity Results of ALPEG Microcapsules

Table 7 gives the total phenolic content (TPC) and DPPH (2,2-diphenyl-1-picrylhydrazyl) scavenging activity results of the ALPEG microcapsules.

AL-Control exhibited a negligible TPC value (0.49 mg GAE·g^−1^ capsule), confirming the absence of phenolic compounds in the shell matrix. In contrast, all ALPEG microcapsules showed markedly higher TPC values. The total phenolic content of ALPEG samples in milligrams of gallic acid equivalent per gram of microcapsule (mg GAE/g microcapsule) was 6.4–66.1 times higher compared to that of the AL-Control. On the other hand, high TPC values obtained for ALPEG-3, ALPEG-5, and ALPEG-6, despite the absence of thyme oil and oleic acid in their core compositions, can be attributed to the inherent limitations of the Folin–Ciocalteu test rather than to the presence of true phenolic compounds [92]. In this context, the Folin–Ciocalteu reagent also responded to the reducing sites of the alginate–chitosan shell matrix, together with PEG1000 and/or PEG1500 incorporated into the shell structure, containing abundant hydroxyl and ether groups [93]. Consequently, higher TPC values were found for ALPEG-10 (17.77 mg GAE·g^−1^) compared to ALPEG-3 (11.52 mg GAE·g^−1^), for ALPEG-11 (12.42 mg GAE·g^−1^) compared to ALPEG-5 (12.11 mg GAE·g^−1^), and for ALPEG-9 (8.15 mg GAE·g^−1^) compared to ALPEG-6 (3.77 mg GAE·g^−1^), indicating the presence of thyme oil phenolic groups in the structure [94,95].

As summarized in Table 7, the DPPH free radical scavenging activities normalized to capsule mass (%·g^−1^) fall within a relatively narrow range for all microcapsule samples, varying between 4.12 and 5.61%·g^−1^. The small differences observed between individual measurements can be attributed to the differences in shell and core compositions as well as the instantaneous capsule formation environment. The AL-Control sample exhibited a remarkable scavenging activity of 4.70%·g^−1^, which can be attributed to the inherent reducing capacity of the alginate–chitosan matrix and confirms that the shell materials themselves contribute measurably to DPPH reduction [96,97]. ALPEG-3, ALPEG-5, and ALPEG-6, which do not contain TO-OA in their cores, displayed scavenging activities comparable to that of AL-Control (4.12–5.61%·g^−1^). This observation shows that the incorporation of PEG1000 and/or PEG1500 into the shell structure does not significantly alter the intrinsic radical scavenging behavior of the alginate–chitosan matrix. However, the incorporation of PEGs into the alginate–chitosan network appears to reduce the relative content and availability of their reactive groups, leading to the relatively lower DPPH free radical scavenging activity observed in ALPEG-5 and ALPEG-6. Meanwhile, ALPEG-11 and ALPEG-9, which contain thyme oil dispersed in oleic acid within their cores, exhibited an improved DPPH scavenging activity, 4.24–5.58%·g^−1^ and 4.80–5.39%·g^−1^ respectively, compared to their shell-only counterparts (4.12 and 4.35%·g^−1^), indicating that the encapsulated TO-OA core slightly contributed to the antioxidant performance of these microcapsules.

Finally, DPPH radical scavenging activity test results were grouped considering the microcapsules without (ALPEG-3, ALPEG-5, ALPEG-6) and with (ALPEG-10, ALPEG-11, ALPEG-9) TO-OA in their cores, and named as Group1 and Group2, respectively. After confirming the homogeneity of variances, an independent two-sample *t*-test was performed to compare the four DPPH measurements of Group 1 with the six measurements of Group 2. Based on the calculated average (AVE) and standard deviation (SD) values (Group1: AVE = 4.70%·g^−1^ and SD = 0.65%·g^−1^; Group2: AVE = 4.89%·g^−1^ and SD = 0.57%·g^−1^), the pooled standard deviation was calculated as 0.60%·g^−1^, and the t-test yielded a t value of −0.48 (t_0_._0258_ = −2.306; α = 0.05). Since the calculated t value fell within the acceptance region of the t-distribution and the corresponding *p* value was greater than 0.05, the contribution of the TO-OA core to the DPPH radical scavenging activity of ALPEG-10, ALPEG-11, and ALPEG-9 was found to be statistically insignificant. However, in the short-term DPPH test period, the diffusion and release of the TO–OA core through the shell containing long PEG chains may have been restricted. This indicates why the differences in antioxidant activity between the TO–OA core-loaded microcapsules and their corresponding core-free counterparts were statistically insignificant. A similar observation was recently reported by Duan et al. (2026) for antioxidant electrospun nanofibers [98].

## 4. Conclusions

In the present study, we successfully developed bio-based and multifunctional shell–core microcapsules via coaxial electrospraying followed by Ca^+2^-induced ionotropic gelation and polyelectrolyte complexation. Accordingly, a total of six types of ALPEG microcapsules were fabricated, comprising three distinct alginate–PEG–chitosan shell compositions, each prepared either with or without a thyme oil–oleic acid core.

SEM analyses confirmed successful microcapsule formation, with morphology influenced by PEG incorporation and core loading. FTIR results verified the presence of alginate, chitosan, and PEG within the shell structure, as well as thyme oil and oleic acid in the core, based on their characteristic infrared transmission bands.

TGA results demonstrated that the presence of PEG improved the thermal stability of the microcapsule with delayed degradation up to 370.0 °C and reduced mass loss relative to PEG-free control. Slightly higher initial mass losses (<118.0 °C) in core-loaded samples were associated with partial evaporation of TO-OA. DSC analyses over ten heating–cooling cycles showed that all ALPEG microcapsules revealed substantial phase transition enthalpies (70.5–91.8 J·g^−1^ at 37.6–48.5 °C), primarily governed by the PCM behavior of PEGs, their incorporation ratios, and thermal storage efficiencies within the shell composition, confirming efficient thermal energy storage–release capability of the microcapsules. DLS measurements (20–55 °C) of aqueous dispersions of microcapsules showed reversible hydrodynamic size variations during thermal cycling, indicating temperature-responsive swelling–deswelling behavior managed by hydrogel–PEG interactions. The presence of the hydrophobic TO-OA core moderately reduced the extent of thermal expansion without preventing reversibility.

All ALPEG microcapsules demonstrated pronounced pH-responsive swelling, significantly greater at pH 7.0 than at pH 3.0. The presence of PEG generally enhanced the water uptake of the microcapsules. Swelling degrees also showed time-dependent increases for all samples at both pH environments. Furthermore, the presence of the hydrophobic TO-OA core moderated swelling by partially limiting water penetration into the matrix. ALPEG microcapsules also exhibited high water solubility within 24 h, consistent with their swelling behavior. TPC and DPPH analyses showed that antioxidant performance arises from both the alginate–chitosan shell and the encapsulated TO-OA core. While PEG incorporation did not significantly alter intrinsic scavenging activity, core-loaded systems exhibited enhanced antioxidant functionality.

Overall, this study provides new insight into the design of bio-based multifunctional microcapsules with heat storage and release capability and reversible thermo-responsive expansion–contraction behavior in the range of 20.0–55.0 °C, pH-responsive tunable swelling and water solubility, and antioxidant activity. Such multifunctional characteristics expand their potential applications in smart food packaging, functional textiles, personal care and cosmetic formulations, and controlled release systems.

## Figures and Tables

**Table 1:** Composition, pH, and conductivity values of the ALPEG microcapsule formulations. (AL(aq) = 1.75 wt% Alginate(aq); CH(aq) = 2.0 wt% Chitosan(aq); TO = Thyme oil; OA = Oleic acid).

	Shell Mixture					Core Mixture				Bath 2				
Sample	AL(aq) (mL)	PEG 1000 (g)	PEG 1500 (g)	pH	Conductivity (mS)	TO (g)	OA (g)	pH	Conductivity (µS)	CH(aq) (mL)	PEG 1000 (g)	PEG 1500 (g)	pH	Conductivity (mS)
AL-Control	25.0	-	-	7.21	3.52	-	-	3.38	0.14	30.0	-	-	4.15	2.54
ALPEG-3	25.0	0.88	-	7.02	3.30	-	-	3.38	0.14	30.0	10.0	-	5.35	0.37
ALPEG-10	25.0	0.88	-	7.02	3.30	0.20	0.44	3.27	0.12	30.0	10.0	-	5.35	0.37
ALPEG-5	25.0	-	0.88	7.40	2.85	-	-	3.38	0.14	30.0	-	10.0	5.23	0.28
ALPEG-11	25.0	-	0.88	7.40	2.85	0.20	0.44	3.27	0.12	30.0	-	10.0	5.23	0.28
ALPEG-6	25.0	0.44	0.44	6.99	2.81	-	-	3.27	0.12	30.0	5.00	5.00	5.63	0.32
ALPEG-9	25.0	0.44	0.44	6.99	2.81	0.20	0.44	3.27	0.12	30.0	5.00	5.00	5.63	0.32

**Table 2:** Coaxial electrospraying parameters for ALPEG shell–core microcapsule production.

Specimen	Shell Mixture Dynamic Viscosity (Pa·s)	Core Mixture Dynamic Viscosity (Pa·s)	Shell Pump Rate (mL·h^−1^)	Core Pump Rate (mL·h^−1^)	Injector Voltage (kV)	Collector Voltage (kV)	Injector–Collector Distance (cm)	Δt (min)
AL-Control	3.65	-	4.00	0.70	(+) 1.04	(−) 1.04	15.0	15
ALPEG-3	3.50	-	4.00	0.70	(+) 1.04	(−) 1.04	15.0	15
ALPEG-10	3.50	0.17	4.00	0.70	(+) 1.04	(−) 1.04	15.0	15
ALPEG-5	3.65	-	4.00	0.70	(+) 1.04	(−) 1.04	15.0	15
ALPEG-11	3.65	0.19	4.00	0.70	(+) 1.04	(−) 1.04	15.0	15
ALPEG-6	4.24	-	4.00	0.70	(+) 1.04	(−) 1.04	15.0	15
ALPEG-9	4.24	0.21	4.00	0.70	(+) 1.04	(−) 1.04	15.0	15

**Table 3:** Thermogravimetry data of ALPEG microcapsules (25.0 °C to 600.0 °C).

Specimen	Mass % at 118.0 °C	Mass% at 156.0 °C	Mass% at 370.0 °C	Mass% at 420.0 °C	Mass% at 520.0 °C
AL-Control	84	81	48	46	41
ALPEG-3	80	79	67	19	15
ALPEG-10	75	74	63	8	6
ALPEG-5	88	86	74	13	7
ALPEG-11	83	81	71	10	7
ALPEG-6	74	73	63	10	7
ALPEG-9	78	77	67	10	6

**Table 4:** Comparative DSC results of ALPEG microcapsules obtained from the 10th heating–cooling cycle.

Sample	10th Heating				10th Cooling			
	Phase Transition (°C)			ΔH (J·g^−1^)	Phase Transition (°C)			ΔH (J·g^−1^)
	T_onset_	T_peak_	T_end_		T_onset_	T_peak_	T_end_	
ALPEG-3	19.1	30.3	37.4	95.3	24.4	14.1	8.2	−92.2
	32.6	41.4	44.0	84.5	23.9/13.7	18.9/11.8	14.4/9.4	−34.4/−17.8
	31.7	38.4	40.5	45.4	19.0	15.6	12.7	−44.4
	27.8 ± 7.6	36.7 ± 5.7	40.6 ± 3.3	75.1 ± 26.3	19.0 ± 5.3	13.8 ± 1.9	10.1 ± 2.3	−62.9 ± 25.7
ALPEG-10	18.0	35.2	40.8	74.4	29.3	23.0	10.9	−66.2
	32.9	41.1	43.4	80.5	24.5	19.2	15.1	−80.7
	25.0	36.5	38.7	56.5	19.5	15.0	10.7	−77.5
	25.3 ± 7.4	37.6 ± 3.1	40.9 ± 2.3	70.5 ± 12.5	24.4 ± 4.9	19.1 ± 4.0	12.3 ± 2.5	−74.8 ± 7.6
ALPEG-5	43.8	49.7	52.5	87.1	27.2	22.1	18.3	−85.7
	35.8	46.3	49.2	112.3	18.7	13.0	8.9	−112.5
	43.6	49.5	52.1	76.0	25.8	22.9	19.5	−80.2
	41.1 ± 4.6	48.5 ± 1.9	51.3 ± 1.8	91.8 ± 18.6	23.9 ± 4.6	19.4 ± 5.5	15.6 ± 5.8	−92.8 ± 17.3
ALPEG-11	42.5	50.3	53.1	76.0	30.3	23.5	18.2	−76.2
	32.1	43.4	47.6	93.0	14.3	7.6	3.2	−94.6
	37.7	45.6	48.0	71.2	22.2	18.3	13.1	−65.9
	37.4 ± 5.2	46.4 ± 3.6	49.6 ± 3.1	80.0 ± 11.4	22.3 ± 8.0	16.5 ± 8.1	11.5 ± 7.6	−78.9 ± 14.5
ALPEG-6	23.4	36.2	41.3	87.4	24.0	13.7	4.6	−90.7
	33.0	45.4	49.5	96.1	26.7	21.4	16.7	−92.4
	33.5	45.3	47.9	63.1	22.4	17.3	12.1	−65.7
	30.0 ± 5.7	42.3 ± 5.3	46.2 ± 4.4	82.2 ± 17.1	24.3 ± 2.2	17.5 ± 3.9	11.1 ± 6.1	−82.9 ± 15.0
ALPEG-9	34.0	46.1	48.6	78.6	24.1	19.1	15.0	−83.0
	33.6	46.2	49.0	75.9	25.3	19.5	13.9	−75.6
	27.4	41.2	43.9	72.9	14.4	7.7	2.2	−78.4
	31.7 ± 3.7	44.5 ± 2.9	47.2 ± 2.8	75.8 ± 2.9	21.3 ± 6.0	15.4 ± 6.7	10.4 ± 7.1	−79.0 ± 3.7

Table 4 includes three individual measurements and their corresponding mean values with standard deviations for each sample.

**Table 5:** pH-dependent swelling degrees (S%) of ALPEG microcapsules.

Sample	Swelling% (pH = 3.0)		Swelling% (pH = 7.0)		Water Solubility% (24 h)	
	t = 30 min	t = 60 min	t = 30 min	t = 60 min	pH = 3.0	pH = 7.0
AL-Control	14.7	40.7	84.5	253.0	86.2	74.9
ALPEG-3	77.3	168.6	175.2	411.9	92.1	96.0
ALPEG-10	31.4	62.8	114.8	372.2	92.0	100.0
ALPEG-5	11.8	25.0	143.6	948.4	84.7	66.8
ALPEG-11	18.7	28.9	36.6	489.0	80.5	61.4
ALPEG-6	27.4	65.8	196.8	394.5	89.3	100.0
ALPEG-9	31.4	71.4	157.1	477.2	90.6	100.0

**Table 6:** The mean hydrodynamic diameter (Z_ave_) data obtained in DLS thermal cycling analyses of ALPEG suspensions, heating from 20.0 °C to 55.0 °C followed by cooling from 55.0 °C to 20.0 °C.

	Z_ave_ (nm)						
T (°C)	AL-Control	ALPEG-3	ALPEG-10	ALPEG-5	ALPEG-11	ALPEG-6	ALPEG-9
20.0	296 ± 34	426 ± 85	456 ± 36	759 ± 16	541 ± 33	572 ± 113	898 ± 11
25.0	313 ± 53	467 ± 136	481 ± 10	887 ± 29	621 ± 53	566 ± 67	948 ± 41
30.0	404 ± 191	645 ± 154	487 ± 27	901 ± 58	656 ± 49	549 ± 71	1096 ± 113
35.0	432 ± 39	727 ± 155	474 ± 12	1136 ± 156	701 ± 7	601 ± 78	1004 ± 199
40.0	630 ± 48	860 ± 108	494 ± 11	1370 ± 156	750 ± 59	605 ± 66	1183 ± 368
45.0	794 ± 90	740 ± 58	524 ± 14	1375 ± 48	726 ± 22	615 ± 55	1078 ± 42
50.0	1047 ± 61	877 ± 70	587 ± 24	1578 ± 82	758 ± 11	593 ± 22	1083 ± 48
55.0	883 ± 97	896 ± 43	691 ± 28	1698 ± 53	879 ± 271	654 ± 8	1124 ± 50
50.0	658 ± 93	782 ± 82	713 ± 30	1904 ± 251	745 ± 73	570 ± 17	1119 ± 98
45.0	539 ± 20	824 ± 102	647 ± 41	1817 ± 128	682 ± 64	595 ± 50	1162 ± 130
40.0	375 ± 38	746 ± 47	689 ± 34	1640 ± 184	640 ± 31	570 ± 53	1058 ± 39
35.0	322 ± 6	682 ± 59	641 ± 29	1386 ± 48	632 ± 31	542 ± 30	1033 ± 69
30.0	288 ± 9	494 ± 54	668 ± 9	1259 ± 43	605 ± 12	530 ± 25	983 ± 30
25.0	268 ± 16	467 ± 118	605 ± 13	1244 ± 63	591 ± 33	496 ± 36	879 ± 59
20.0	276 ± 14	424 ± 60	621 ± 24	1313 ± 56	627 ± 13	526 ± 20	893 ± 29
I	3.0	2.1	1.5	2.2	1.6	1.1	1.3
II	3.2	2.1	1.1	1.3	1.4	1.2	1.3

I: max expansion ratio on heating; II: max contraction ratio on cooling.

**Table 7:** Total phenolic content (TPC) and DPPH free radical scavenging activities of ALPEG microcapsules.

Sample	TPC ^1^ (mg GAE·g^−1^ Capsule)	DPPH Scavenging Activity (%·g^−1^ Capsule)
AL-Control	0.49	4.70
ALPEG-3	11.52	4.71–5.61
ALPEG-10	17.77	4.24–5.06
ALPEG-5	12.11	4.12
ALPEG-11	12.42	4.24–5.58
ALPEG-6	3.77	4.35
ALPEG-9	8.15	4.80–5.39

^1^ Gallic acid equivalent (GAE).

## Data Availability

The original contributions presented in this study are included in the article/Supplementary Material. Further inquiries can be directed to the corresponding authors.

## Supplementary Materials

The following supporting information can be downloaded at https://www.mdpi.com/article/10.3390/polym18172082/s1. Supplementary S1: FTIR Spectra of Sodium Alginate and Chitosan. Figure S1. FTIR spectra of Na-alginate (AL) and chitosan (CH). Table S1. FTIR band assignments of Na-alginate and chitosan. Supplementary S2: The FTIR spectra of PEG1000 and PEG1500. Figure S2. The FTIR spectra of PEG1000 and PEG1500. Table S2. FTIR band assignments of PEG1000 and PEG1500. Supplementary S3: The FTIR spectra of thyme oil and oleic acid. Figure S3. The FTIR spectra of thyme oil and oleic acid. Table S3. FTIR spectral band assignments for thyme oil and oleic acid. Supplementary S4: DLS (Dynamic Light Scattering) thermal cycling analyses of aqueous. Figure S4. The mean hydrodynamic diameter (Zave) data of the suspensions of: (a) AL-Control, (b) ALPEG-3 and ALPEG-10, (c) ALPEG-5 and ALPEG-15, (d) ALPEG-6 and ALPEG-9 in DLS thermal cycling analyses, heating from 20.0 °C to 55.0 °C followed by cooling from 55.0 °C to 20.0 °C. Table S4. The polydispersity index data (PDIs) of the ALPEG suspensions between 20.0 °C and 55.0 °C. Supplementary S5: Differential Scanning Calorimeter (DSC) heating curve of Thyme Oil from 20 °C to 300 °C. Figure S5. DSC heating curve of thyme oil from 20 °C to 300 °C.
