# Supplementary material for: Preparation of Multifunctional Alginate–PEG–Chitosan Double Shell and Thyme Oil–Oleic Acid Core Microcapsules via Coaxial Electrospraying
Source: Polymers (Basel). 2026 Aug 27;18(17):2082. doi: 10.3390/polym18172082 (PMC13568044; doi:10.3390/polym18172082)
Supplement: Supplementary file 1 [file polymers-18-02082-s001.zip › polymers-4454023-supplementary.pdf]

Preparation of Multifunctional Alginate–PEG–Chitosan Double Shell and Thyme Oil–Oleic Acid Core Microcapsules via Coaxial Electrospraying

Emel Onder [1,*], Sena Saritop [2] and Nihal Sarier [2,3,*]

[1] Faculty of Textile Technologies and Design, Istanbul Technical University, Gumussuyu, Istanbul 34437, Türkiye
[2] Faculty of Engineering, Istanbul Kültür University, Atakoy Campus, Bakirkoy, Istanbul 34156, Türkiye
[3] Spectroscopy@IKU, IKU-SPECTRA Molecular Sciences and Spectroscopy Applied Research Center, Istanbul Kültür University, Atakoy Campus, Bakırkoy, Istanbul 34156, Türkiye
* Correspondence: onderem@itu.edu.tr (E.O.); n.sarier@iku.edu.tr (N.S.)

Contents: (Number of pages=12)

Supplementary S1: FTIR Spectra of Sodium Alginate and Chitosan

Supplementary S2: The FTIR spectra of PEG1000 and PEG1500

Supplementary S3: The FTIR spectra of thyme oil and oleic acid

Supplementary S4: DLS (Dynamic Light Scattering) thermal cycling analyses of aqueous suspensions of ALPEG microcapsules, heating from 20.0 °C to 55.0 °C followed by cooling from 55.0 °C to 20.0 °C

Supplementary S5: Differential Scanning Calorimeter (DSC) heating curve of Thyme Oil from 20 °C to 300 °C

Supplementary S1: FTIR Spectra of Sodium Alginate and Chitosan

The FTIR spectrum of sodium alginate displays all characteristic bands of a typical anionic polysaccharide. The broad transmission band centered at 3265 cm^{-1}, corresponding to –OH stretching vibrations (ν_{O-H}), is indicative of inter- and intramolecular hydrogen bonding (Ramesh Babu et al.,2006). The band at 2921 cm^{-1} represents C–H stretching vibrations (ν_{C-H}) of aliphatic chains.

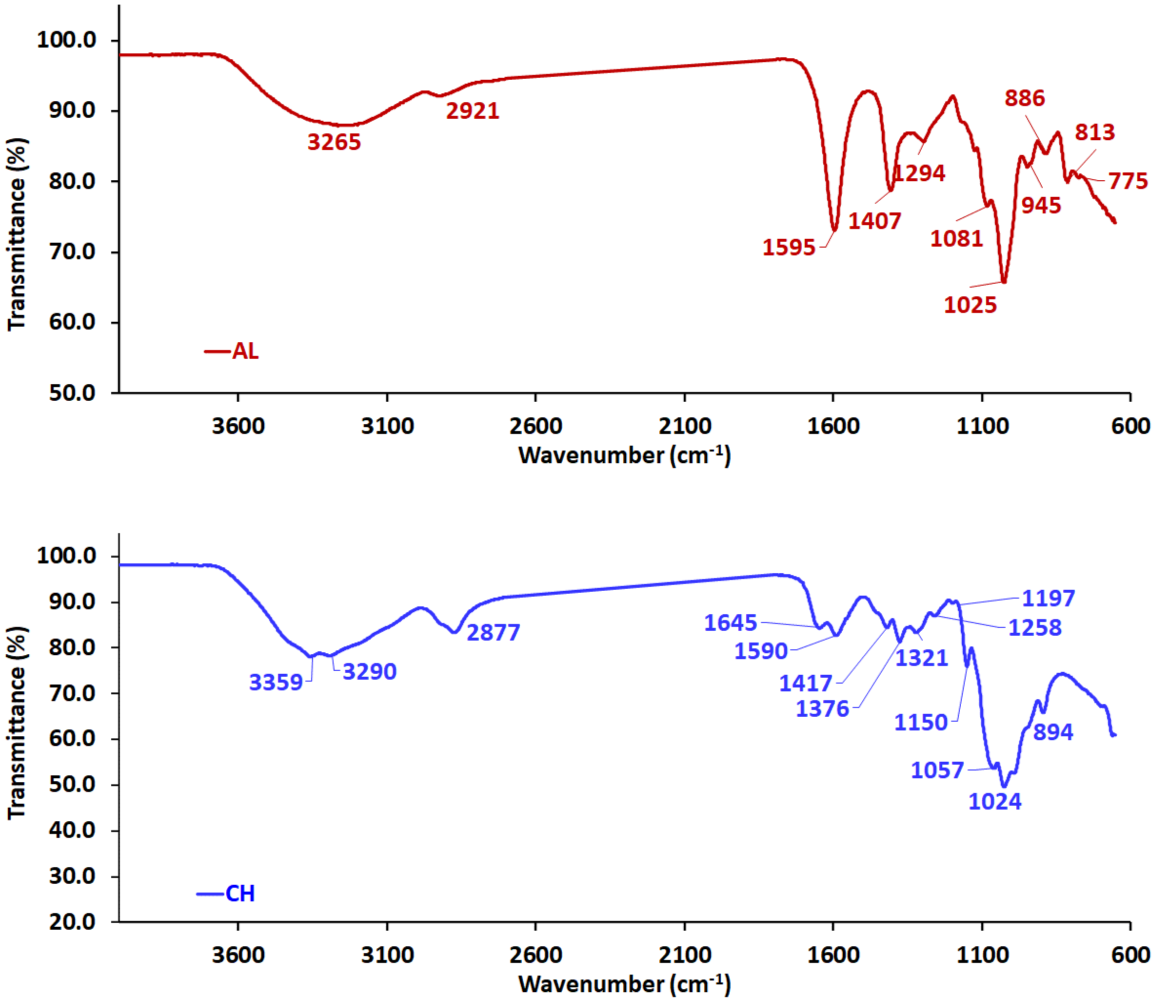

Figure S1. FTIR spectra of Na-Alginate (AL) and Chitosan (CH).

The strong bands at 1595 cm^{-1} and 1407 cm^{-1} indicate the presence of carboxylate (COO^-) groups in asymmetric and symmetric stretching modes, respectively, confirming its identity as a salt form (Na^+) of alginic acid (George & Abraham, 2006; Papageorgiou et al., 2010). The band around 1294 cm^{-1} is attributed to C–O stretching in the uronic acid residues. Additionally, the small band at 1079 cm^{-1} and the strong band at 1025 cm^{-1} are assigned to C–O–C stretching vibrations(ν_{C-O}) (Papageorgiou et al., 2010). The 945 cm^{-1} band is typically

associated with glycosidic and ring structures of mannuronic and guluronic acid ring vibrations (Pereira et al., 2009).

FTIR bands at 886, 813, and 775 cm^{-1} are linked to ring deformations and skeletal vibrations of the alginate pyranose ring. In addition, the band at 813 cm^{-1} is attributed to Na-O bond in Na-alginate (Şolpan et al., 2008). These features verify the polymeric saccharide nature and ionic functionality of sodium alginate (Bekin et al., 2014). The FTIR spectrum of chitosan is characterized by a broad band at 3359 cm^{-1} with a shoulder at 3290 cm^{-1}, attributed to overlapping O–H and N–H stretching vibrations, due to extensive hydrogen bonding (Rinaudo, 2006). The absorption at 2877 cm^{-1} is due to C–H stretching of the aliphatic groups. The 1645 cm^{-1} band corresponds to amide I (C=O stretching) of N-acetyl groups, while 1590 cm^{-1} arises from N–H bending (amide II) of primary amines. Bands at 1417 cm^{-1} and 1376 cm^{-1} are assigned to CH_2 scissoring and CH_3 bending, respectively. The 1150 cm^{-1} band is indicative of asymmetric stretching of the C–O–C bonds between saccharide units. Strong C–O stretching vibrations appear at 1024 and 1057 cm^{-1}, and the band at 894 cm^{-1} is due to β-glycosidic linkages between sugar units (Kasaai, 2009) (Duarte et al., 2002).

Table S1. FTIR Band Assignments of Na Alginate and Chitosan.

Specimen	Wavenumber(cm^{-1})	Functional Group Assignment
**Na Alginate**	3265	O–H stretching (H-bonded)
	2921	C–H stretching
	1595	COO^- asymmetric stretching
	1407	COO^- symmetric stretching
	1294	C–O stretching
	1025	C–O–C stretching
	945	Saccharide ring vibration
**Chitosan**	3359, 3290	O–H and N–H stretching (H-bonded)
	2877	C–H stretching
	1645	Amide I (C=O stretching)
	1590	N–H bending (amide II)
	1417	CH_2 deformation
	1376	CH_3 bending
	1150	C–O–C asymmetric stretching
	1057, 1024	C–O stretching
	894	β-glycosidic linkage

References

Bekin, S., Sarmad, S., Gürkan, K., Yenici, G., Keçeli, G., & Gürdağ, G. (2014). Dielectric, thermal, and swelling properties of calcium ion-crosslinked sodium alginate film. *Polym Eng Sci.*, 54(6), 1372-1382. https://doi.org/10.1002/pen.23678

Duarte, M. L., Ferreira, M. C., Marvao, M. R., & Rocha, J. (2002). An optimised method to determine the degree of acetylation of chitin and chitosan by FTIR spectroscopy. *Int J Biol Macromol.*, 31(1-3), 1-8. https://doi.org/10.1016/S0141-8130(02)00039-9

George, M., & Abraham, T. E. (2006). Polyionic hydrocolloids for the intestinal delivery of protein drugs: alginate and chitosan—a review. *J Control Release*, 114(1), 1-14. https://doi.org/10.1016/j.jconrel.2006.04.017

Kasaai, M. R. (2009). Various methods for determination of the degree of N-acetylation of chitin and chitosan: A review. *J Agric Food Chem.*, 57(5), 1667–1676. https://doi.org/10.1021/jf803001m

Papageorgiou, S. K., Kouvelos, E. P., Favvas, E. P., Sapalidis, A. A., Romanos, G. E., & Katsaros, F. K. (2010). Metal–carboxylate interactions in metal–alginate complexes studied with FTIR spectroscopy. *Carbohydr Res.*, *345*(4), 469-473. https://doi.org/10.1016/j.carres.2009.12.010

Pereira, L., Amado, A. M., Critchley, A. T., Van de Velde, F., & Ribeiro-Claro, P. J. (2009). Identification of selected seaweed polysaccharides (phycocolloids) by vibrational spectroscopy (FTIR-ATR and FT-Raman). *Food Hydrocoll.*, 23(7), 1903-1909.https://doi.org/10.1016/j.foodhyd.2008.11.014

Ramesh Babu, V., Krishna Rao, K. S. V., Sairam, M., Naidu, B. V. K., Hosamani, K. M., & Aminabhavi, T. M. (2006). pH sensitive interpenetrating network microgels of sodium alginate-acrylic acid for the controlled release of ibuprofen. *J Appl Polym Sci.*, 99(5), 2671-2678. https://doi.org/10.1002/app.22760

Rinaudo, M. (2006). Chitin and chitosan: Properties and applications. *Prog Poly Sci.*, 31(7), 603–632. https://doi.org/10.1016/j.progpolymsci.2006.06.001

Şolpan, D., Torun, M., & Güven, O. (2008). The usability of (sodium alginate/acrylamide) semi-interpenetrating polymer networks on removal of some textile dyes. *J Appl Polym Sci.*, 108(6), 3787-3795. https://doi.org/10.1002/app.27945

Supplementary S2: The FTIR spectra of PEG1000 and PEG1500

The FTIR spectra of PEG1000 and PEG1500 exhibit typical features characteristic of polyethylene glycol chains, primarily dominated by the vibrational modes of the ethylene oxide repeat units and terminal hydroxyl groups. A broad transmission band around 3449 cm^{-1} for PEG1000 and 3415 cm^{-1} for PEG1500 corresponds to the O–H stretching vibrations, indicative of hydroxyl end groups and hydrogen bonding (Silverstein et al., 2014). Both spectra exhibit C–H stretching at 2883 cm^{-1}, arising from the $–CH_2–$ groups in the polymer backbone (Onder et al., 2013). The weak C=O stretching vibration at 1746 cm^{-1} may be attributed to trace ester linkages or oxidative degradation products (Deygen & Kudryashova, 2016).

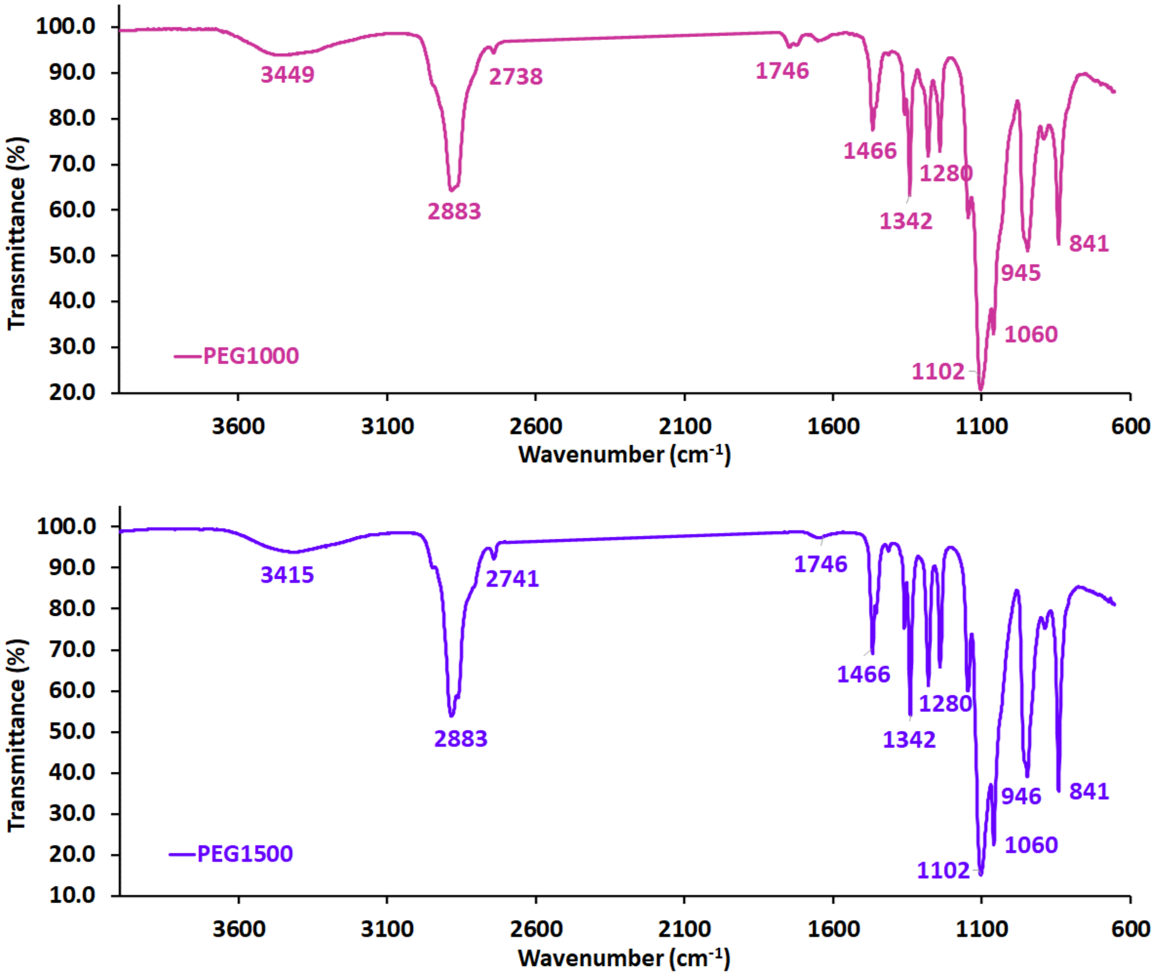

Figure S2. The FTIR spectra of PEG1000 and PEG1500.

Bands at 1466 cm^{-1}, 1342 cm^{-1}, and 1280 cm^{-1} correspond to CH_2 bending and wagging vibrations, reflecting the flexible backbone of PEG chains (Onder et al., 2013). The most prominent features are observed in the fingerprint region, with intense bands at 1102 cm^{-1} and

1060 cm⁻¹, assigned to the C–O–C asymmetric and symmetric stretching modes of ether bonds (Noyan et al., 2018). Minor bands at 945 cm^{-1} and 841 cm^{-1} represent C–H out-of-plane bending. Notably, PEG1000 and PEG1500 show similar spectral profiles, with subtle differences in band broadness and intensity due to variations in molecular weight and the proportion of terminal functional groups (Deygen & Kudryashova, 2016).

Table S2. FTIR Band Assignments of PEG1000 and PEG1500.

Wavenumber (cm^{-1})	PEG1000	PEG1500	Functional Group Assignment
3449-3415	✓	–	O–H stretching (hydrogen bonded –OH end groups)
2883-2741-2738	✓	✓	C–H stretching (–CH_2–) &Weak overtone/combination bands
1746	✓	✓	C=O stretching (traces of ester)
1466	✓	✓	CH_2 scissoring (bending)
1342	✓	✓	CH_2 wagging
1280	✓	✓	CH_2 twisting/wagging
1102	✓	✓	C–O–C stretching (ether group)
1060	✓	✓	C–O–C symmetric stretching
945	✓	✓	C–H out-of-plane bending
841	✓	✓	C–H deformation (PEG chain)

References:

Deygen, I. M., & Kudryashova, E. V. (2016). New versatile approach for analysis of PEG content in conjugates and complexes with biomacromolecules based on FTIR spectroscopy. *Colloids Surf B.*, *141*, 36-43. https://doi.org/10.1016/j.colsurfb.2016.01.030

Noyan, E. C., Onder, E., Sarier, N., & Arat, R. (2018). Development of heat storing poly (acrylonitrile) nanofibers by coaxial electrospinning. *Thermochim Acta.*, 662, 135-148. https://doi.org/10.1016/j.tca.2018.02.008

Onder, E., Sarier, N., Ukuser, G., Ozturk, M., & Arat, R. (2013). Ultrasound assisted solvent free intercalation of montmorillonite with PEG1000: A new type of organoclay with improved thermal properties. *Thermochim Acta.*, 566, 24-35. https://doi.org/10.1016/j.tca.2013.05.021

Silverstein, R.M., Webster F.X., Kiemle D.J. and Bryce D.L. (2015) Spectrometric Identification of Organic Compounds. 8th Edition, *John Wiley & Sons, Ltd*. Chi-chester, 230-298. https://doi.org/10.1021/ed039p546

Supplementary S3: The FTIR spectra of thyme oil and oleic acid

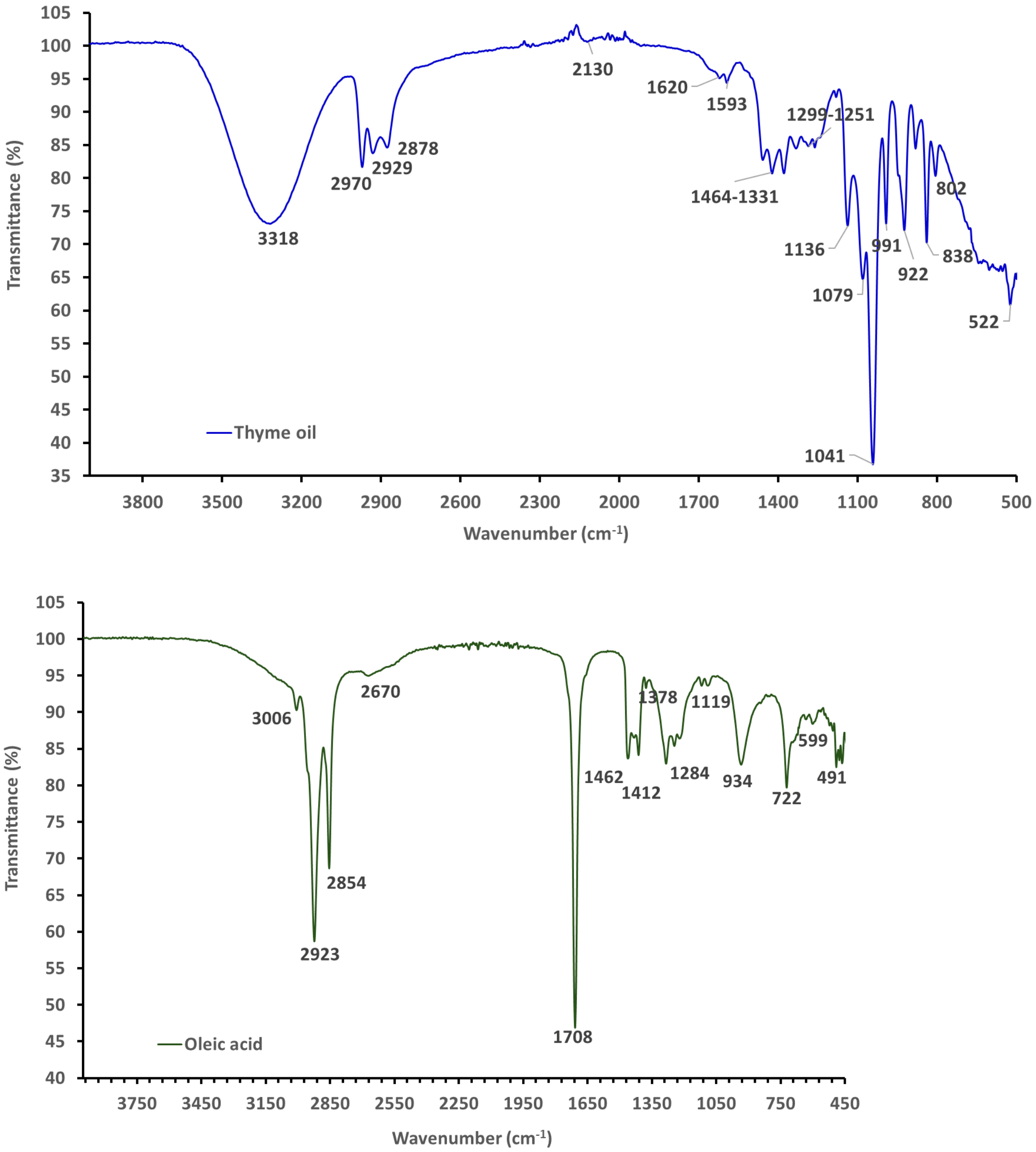

Figure S3. The FTIR spectra of thyme oil and oleic acid.

The FTIR spectra of thyme oil and oleic acid reveal distinct chemical signatures reflecting their unique molecular structures. Thyme oil exhibits characteristic transmission bands indicative of its aromatic and phenolic constituents, such as thymol, carvacrol, and p-cymene. A broad absorption band at 3318 cm^{-1} confirms the presence of phenolic –OH groups from thymol and carvacrol via hydrogen-bonded O–H stretching (Abbad et al., 2026), while the strong bands stretching across 2970–2878 cm^{-1} correspond to the symmetric and asymmetric C–H stretching

of aliphatic –CH_3 and –CH_2 groups shared across the oil's monoterpene backbone (Burt, 2004; Soković et al., 2010). . The aromatic core of these compounds is verified by a weak 2130 cm^{-1} overtone combination band alongside prominent C=C ring stretching vibrations at 1620 cm^{-1} and 1593 cm^{-1}. The band at 1411 cm^{-1} serves as a characteristic marker of in-plane O–H bending of phenolic groups, reflecting the presence of phenolic constituents such as thymol. Furthermore, the bands at 1457–1331 cm^{-1} correspond to symmetric –CH_3 bending of isopropyl groups, confirming the branched-chain structure typical of the essential oil components (Soni et al., 2022). Additional bands at 1284 cm^{-1}, 1238 cm^{-1}, 1223 cm^{-1} and 1162 cm^{-1} are attributed to C–O–C stretching in ether or phenolic structures, and strong bands in the range of 936 to 722 cm^{-1} reflect aromatic ring deformation and C–H bending vibrations, characteristic of substituted aromatic compounds (Kokina et al., 2015). Finally, the structural geometry and specific substitution patterns of the benzene ring units are established in the lower fingerprint region, where in-plane C–H bending occurs at 1172–1041 cm^{-1}, complemented by complex olefinic and monoterpene =C–H out-of-plane bending and ring modes at 991–805 cm^{-1}, and finished by terminal out-of-plane ring skeletal deformations at 642 cm^{-1} and 522 cm^{-1}.

Oleic acid, a monounsaturated fatty acid, displays characteristic FTIR bands typical of long-chain carboxylic acids. The presence of its characteristic cis-alkene (C=C) double bond is verified by the sharp band at 3006–3008 cm^{-1} corresponding to the stretching vibration of the cis C=C double bond within the oleic acid chain (Soni et al., 2022). The aliphatic backbone of oleic acid is evidenced by strong –CH_2 asymmetric and symmetric stretching bands at 2923 and 2854 cm^{-1}, respectively. The acidic head group of the molecule is prominently displayed via a broad O–H stretching shoulder at 2670 cm^{-1} and a sharp, prominent band at 1708 cm^{-1} that corresponds to the C=O stretching vibration of the carboxylic acid group (Silverstein et al., 2014). Aliphatic C–H bending deformations appear at 1462 cm^{-1} and 1378 cm^{-1}, a distinct carboxylic C–O–H in-plane bend registers at 1412 cm^{-1}, and C–O single bond stretching appears at 1284 cm^{-1} and 1223 cm^{-1}. In the fingerprint region, where C–C skeletal shifts emerge between 1136–1089 cm^{-1}, dimeric carboxylic acid loops exhibit O–H out-of-plane wagging at 934 cm^{-1}, and a sharp –CH_2– rocking peak at 722 cm^{-1} confirms a long sequence of adjacent methylene groups, backed by terminal skeletal bending modes from 694–602 cm^{-1}. (Silverstein et al., 2014).

Table S3. FTIR spectral band assignments for thyme oil and oleic acid.

Sample	Wavenumber (cm^{-1})	Vibrational Mode	Functional Group / Assignment
**Thyme Oil**	3318	Broad O–H stretching	Phenolic –OH groups(Thymol, Carvacrol)
	2970-2878	.CH_3 and CH_2 symmetric asymmetric stretching	Aliphatic –CH_3, –CH_2 groups (Monoterpenes)
	2130	Weak aromatic overtone/combination band	Benzene ring structures
	1620, 1593	C=C stretching, aromatic ring stretching	Aromatic C=C bonds (Thymol, Carvacrol, p-Cymene)
	1457-1331	CH_2/CH_3 bending (scissoring)	Isopropyl side chains
	1284, 1223	C–O–C and C–O stretching	Ether, phenolic –OH groups (Thymol, Carvacrol)
	1172, 1136, 1079, 1041	In-plane aromatic C–H bending	Benzene ring structures
	991, 922,880, 838, 805	=C–H out-of-plane bending/ring modes	Olefinic groups, monoterpenes, aromatic rings
	642, 522	Aromatic ring out-of-plane skeletal deformation	Thymol/Carvacrol derivatives
**Oleic Acid**	3006-3008	=C–H stretching	Cis-double bond in unsaturated oleic acid
	2923, 2854	CH_2 symmetric, asymmetric stretching	Long-chain aliphatic –CH_2
	2670	Broad carboxylic O-H stretch	Carboxylic acid dimer formation
	1708	C=O stretching	Carboxylic acid group (-COOH)
	1462, 1378	C–H aliphatic bending	Methyl and methylene deformations
	1412	C–O–H in-plane bend	Carboxylic acid group (-COOH)
	1284, 1223	C–O single bond stretch	Carboxylic acid group (-COOH)
	1136 - 1089	C–C skeletal stretch	Hydrocarbon chain backbone
	934	O–H out-of-plane wagging	Carboxylic acid dimer loop
	722	CH_2 rocking	Long methylene chain sequence

References:

Bozin, B., Mimica-Dukić, N., Simin, N., & Anackov, G. (2006). Characterization of the volatile composition of essential oils of some Lamiaceae spices and the antimicrobial and antioxidant

activities of the entire oils. *J Agric Food Chem.*, 54(5), 1822–1828. https://doi.org/10.1021/jf051922u

Burt, S. (2004). Essential oils: their antibacterial properties and potential applications in foods—a review. *Int J Food Microbiol.*, 94(3), 223–253. https://doi.org/10.1016/j.ijfoodmicro.2004.03.022

Abbad, Imane and El Hakioui, Imane and Soulaimani, Bouchra and Miloudi, Soukaina and Drissi, BadrEddine and Taarji, Noamane and Abbad, Abdelaziz and Sobeh, Mansour and Barakate, Mustapha (2026) Optimized Citrus limon/Thymus leptobotrys essential oil nanoemulsion reinforced into pectin/chitosan edible coatings for improving turkey meat shelf-life. Available at SSRN: https://ssrn.com/abstract=6890379 or http://dx.doi.org/10.2139/ssrn.6890379

Kokina, M., Salević, A., Kalušević, A., Lević, S., Pantić, M., Pljevljakušić, D., ... & Nedović, V. (2019). Characterization, antioxidant, and antibacterial activity of essential oils and their encapsulation into biodegradable material, followed by freeze drying. *Food Technol Biotechnol.*, *57*(2), 282. https://doi.org/10.17113/ftb.57.02.19.5957

Silverstein, R.M., Webster F.X., Kiemle D.J. and Bryce D.L. (2015) Spectrometric Identification of Organic Compounds. 8th Edition, *John Wiley & Sons, Ltd*. Chi-chester, 230-298. https://doi.org/10.1021/ed039p546

Soković, M., Glamočlija, J., Marin, P. D., Brkić, D., & van Griensven, L. J. L. D. (2010). Antibacterial effects of the essential oils of commonly consumed medicinal herbs using an in vitro model. *Molecules*, 15(11), 7532–7546. https://doi.org/10.3390/molecules15117532

Soni, A., Yusuf, M., Mishra, V. K., & Beg, M. (2022). An assessment of thermal impact on chemical characteristics of edible oils by using FTIR spectroscopy. *Mater Today Proc.*, 68, 710-716.https://doi.org/10.1016/j.matpr.2022.05.568

Supplementary S4: DLS (Dynamic Light Scattering) thermal cycling analyses of aqueous suspensions of ALPEG microcapsules, heating from 20.0 °C to 55.0 °C followed by cooling from 55.0 °C to 20.0 °C

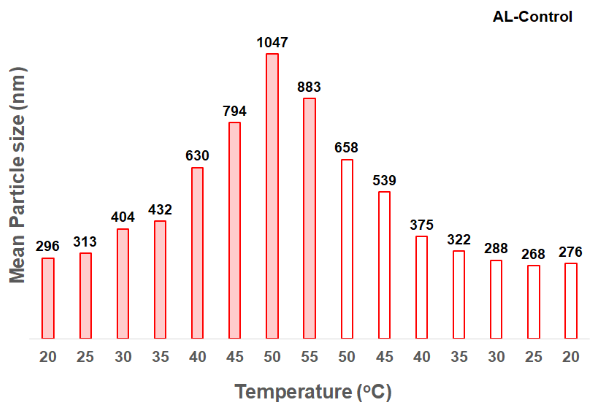

(a)

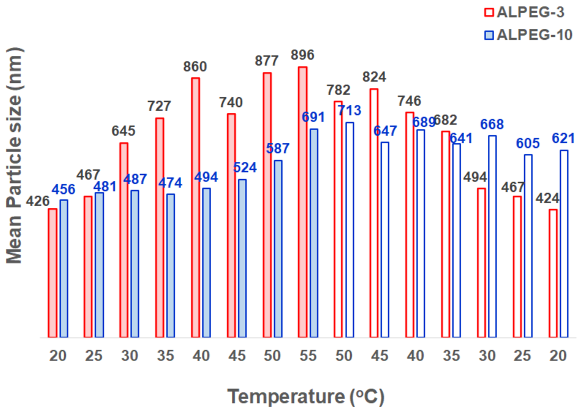

(b)

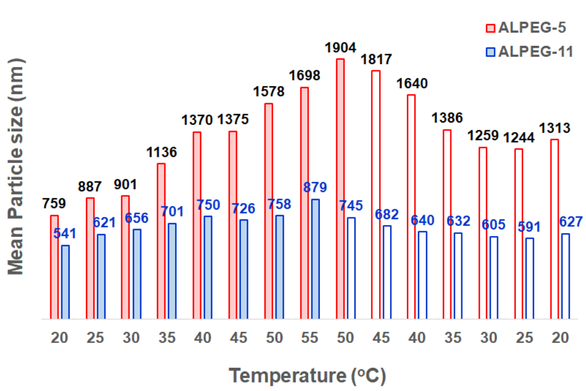

(c)

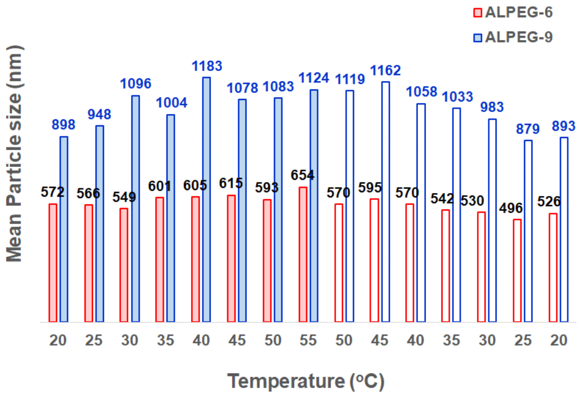

(d)

Figure S4. The mean hydrodynamic diameter (Z_{ave}) data of the suspensions of: a) AL-Control, b) ALPEG-3 and ALPEG-10, c) ALPEG-5 and ALPEG-15, d) ALPEG-6 and ALPEG-9 in DLS thermal cycling analyses, heating from 20.0 °C to 55.0 °C followed by cooling from 55.0 °C to 20.0 °C.

Table S4. The polydispersity index data (PDIs) of the ALPEG suspensions between 20.0 °C and 55.0 °C.

T (°C)	AL-Control	ALPEG-3	ALPEG-10	ALPEG-5	ALPEG-11	ALPEG-6	ALPEG-9
20.0	0.549±0.045	0.620±0.124	0.361±0.058	0.609±0.254	0.525±0.114	0.585±0.194	0.504±0.160
25.0	0.472±0.093	0.642±0.155	0.336±0.068	0.567±0.297	0.551±0.162	0.518±0.129	0.579±0.060
30.0	0.618±0.116	0.797±0.138	0.369±0.097	0.755±0.029	0.605±0.043	0.566±0.143	0.732±0.136
35.0	0.595±0.051	0.812±0.047	0.418±0.017	0.921±0.034	0.595±0.008	0.597±0.120	0.819±0.180
40.0	0.683±0.016	0.889±0.099	0.499±0.044	1.000±0.000	0.701±0.044	0.613±0.079	0.844±0.145
45.0	0.822±0.085	0.815±0.015	0.519±0.017	0.998±0.003	0.634±0.036	0.610±0.065	0.752±0.029
50.0	0.973±0.047	0.873±0.014	0.644±0.040	1.000±0.000	0.656±0.022	0.590±0.055	0.785±0.013
55.0	0.868±0.033	0.953±0.041	0.706±0.047	1.000±0.000	0.693±0.059	0.632±0.066	0.762±0.028

Supplementary S5: Differential Scanning Calorimeter (DSC) heating curve of Thyme Oil from 20 °C to 300 °C

In the heating cycle of the DSC analysis, it is seen that the TO starts to evaporate around 109 °C, reaches its maximum at 153.2 °C and ends at 155.8 °C. The total absorbed heat was calculated 790.4 J.g^{-1}. Results are in good agreement with the data obtained by Kobacy et al. (2025)

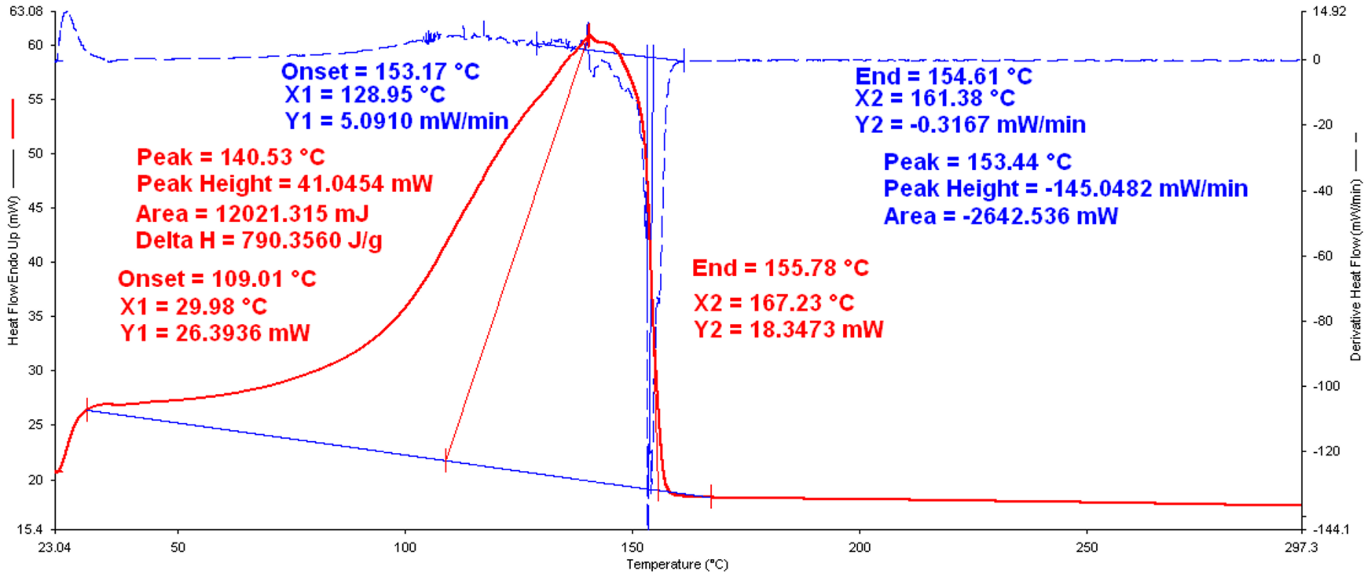

Figure S5. DSC heating curve of Thyme Oil from 20 °C to 300 °C.

References

Kobacy, W., Gong, X., Elshamandy, N. K., Zahran, H. A., Osman, A. I., & Rashwan, A. K. (2025). ATR-FTIR, DSC, GC–MS of garlic (Allium sativum), parsley (Petroselinum crispum), and thyme (Thymus vulgaris) essential oils and their synergistic effects on antioxidant and antimicrobial properties. *Food Bioscience*, 107262.
